# Hordein Accumulation in Developing Barley Grains

**DOI:** 10.3389/fpls.2019.00649

**Published:** 2019-05-16

**Authors:** Gregory J. Tanner, Michelle L. Colgrave, Malcolm J. Blundell, Crispin A. Howitt, Antony Bacic

**Affiliations:** ^1^School of Biosciences, University of Melbourne, Melbourne, VIC, Australia; ^2^Commonwealth Scientific and Industrial Research Organisation Agriculture and Food, St Lucia, QLD, Australia; ^3^School of Science, Edith Cowan University, Joondalup, WA, Australia; ^4^Commonwealth Scientific and Industrial Research Organisation Agriculture and Food, Canberra, ACT, Australia; ^5^La Trobe Institute for Agriculture and Food, La Trobe University, Bundoora, VIC, Australia

**Keywords:** hordeins, accumulation, developing barley grain, multiple reaction monitoring mass spectrometry, gluten

## Abstract

The temporal pattern of accumulation of hordein storage proteins in developing barley grains was studied by enzyme-linked immunosorbent assay (ELISA), western blot and liquid chromatography tandem mass spectrometry (LC-MS/MS). Hordein accumulation was compared to the pattern seen for two abundant control proteins, serpin Z4 (an early accumulator) and lipid transferase protein (LTP1, a late accumulator). Hordeins were detected from 6 days post-anthesis (DPA) and peaked at 30 DPA. Changes in fresh weight indicate that desiccation begins at 20 DPA and by 37 DPA fresh weight had decreased by 35%. ELISA analysis of hordein content, expressed on a protein basis, increased to a maximum at 30 DPA followed by a 17% decrease by 37 DPA. The accumulation of 39 tryptic and 29 chymotryptic hordein peptides representing all classes of hordein was studied by LC-MS/MS. Most peptides increased to a maximum at 30 DPA, and either remained at the maximum or did not decrease significantly. Only five tryptic peptides, members of the related B1- and γ1-hordeins decreased significantly by 21–51% at 37 DPA. Thus, the concentration of some specific peptides was reduced while remaining members of the same family were not affected. The N-terminal signal region was removed by proteolysis during co-translation. In addition to a suite of previously characterized hordeins, two novel barley B-hordein isoforms mapping to wheat low molecular weight glutenins (LMW-GS-like B-hordeins), and two avenin-like proteins (ALPs) sharing homology with wheat ALPs, were identified. These identified isoforms have not previously been mapped in the barley genome. Cereal storage proteins provide significant nutritional content for human consumption and seed germination. In barley, the bulk of the storage proteins comprise the hordein family and the final hordein concentration affects the quality of baked and brewed products. It is therefore important to study the accumulation of hordeins as this knowledge may assist plant breeding for improved health outcomes (by minimizing triggering of detrimental immune responses), nutrition and food processing properties.

## Introduction

Prolamins, are rich in proline and glutamine residues, and this is a collective name given to the alcohol-soluble, water-insoluble storage proteins that exist in wheat gluten (gliadin and glutenin), barley (hordein), rye (secalins), and oat (avenin). The barley hordeins consist of four closely related protein families that are categorized by molecular weight: the D-hordeins, a 105 kDa protein family coded for by a single gene with up to five post-translationally modified isoforms (Gu et al., 2003); the C-hordeins which are 55 and 65 kDa sulfur-poor proteins, coded for by 20–30 genes (Shewry et al., 1985a,b); the B-hordeins which are a group of sulfur-rich proteins running at 50 kDa, coded for by at least 13 genes, with at least two protein sub-families, the B1- and B3-hordeins (Kreis et al., 1983; Anderson, 2013); and the sulfur-rich gamma (γ)-hordeins comprising at least three isoforms of 35–45 kDa. The four hordein protein families are coded for by Hor-1 (C-hordeins), Hor-2 (B-hordeins), Hor-3 (D-hordeins), and Hor-4 (γ-hordeins) loci, located on barley chromosome 1H. The B-hordeins account for 70–90% of total hordeins; the C-hordeins form 10–30% of the hordein fraction; the gamma-hordeins and the D-hordeins are minor components accounting for 1–2, and 2–4%, respectively, of the hordeins (Shewry et al., 1985b).

The hordeins are amongst the triggers of coeliac disease (CD), a well characterized T-cell mediated disorder suffered by approximately 1% of most populations (Fasano et al., 2003; Lebwohl et al., 2018). In CD the immune system mounts an inappropriate reaction to particular peptide sequences in dietary gluten, reacting as if the gluten molecules were an invading microorganism (Anderson et al., 2000). Life-long avoidance of gluten remains the only treatment option for coeliacs. Other adverse reactions to gluten also exist including gluten intolerance (Biesiekierski et al., 2011, 2013; Choung et al., 2017), which affects approximately 10% of the population (Golley et al., 2015), and gluten allergy, a serious, rapid, IgE mediated allergy impacting 1% of the population (Snegaroff et al., 2006).

Hordeins accumulate in the starchy endosperm cells of developing barley grains, during grain filling (Roustan et al., 2018). Hordein synthesis proceeds linearly from approximately 10 to 30 days post-anthesis (DPA) (Brandt, 1976; Sorensen et al., 1989). Hordeins are coordinately expressed on the polyribosomes of the rough endoplasmic reticulum (RER) of starchy endosperm cells, and ∼20 amino acid N-terminal transit peptides are removed during co-translational processing and transport into the ER lumen (Cameron-Mills et al., 1978). Hordeins are then transported from the ER lumen to the central vacuole during maturation (Rechinger et al., 1993; Cameron-Mills et al., 1994; Ibl et al., 2014). The pathway taken by hordeins, from protein body to vacuole is not clear. It has been proposed that hordeins either pass through or by-pass the Golgi network (Bechtel et al., 1991; Muntz, 1998; Gomord et al., 1999; Bethke and Jones, 2000; Pereira et al., 2014).

Gluten proteins have traditionally been measured using ELISA, however, great care must be taken to match the calibration standard with the protein that is being measured (Tanner et al., 2013a). While ELISA may be suitable for total gluten determination in either unprocessed grain or raw ingredients, multiple reaction monitoring mass spectrometry (MRM-MS) is capable of identifying and quantifying individual gluten proteins (Tanner et al., 2013b). MRM-MS is a method where prototypic peptides representing all hordein families are detected and quantified (Colgrave et al., 2012, 2014, 2015, 2016b). In this study we have used both ELISA and MRM-MS to study the pattern of hordein accumulation during grain development to further our understanding of hordein function *in planta.* A fundamental understanding of hordein accumulation in the grain is required for plant breeding applications that aim to either improve the nutritional status of barley or reduce the coeliac reactivity, but may also apply to food and beverage processing.

## Materials and Methods

### Plant Material

Barley cv Sloop was obtained from the Australian Grains Genebank, Department of Environment and Primary Industries (DEPI) Horsham, and germinated in a 50/50 (v/v) mixture of soil (Debco seed raising mix, Tyabb Victoria) and perlite, three plants per 20 cm pot, and grown at constant temperature of 19–24°C, under ambient light with 12 h daylight extension provided by 1500 W halogen lights at 400 μE for approximately 8 weeks until flowering. Plants were watered with a balanced nutrient solution (250 mL per pot of 2.5 g/L Aquasol, Yates Australia Padstow) once every week. Anthesis was determined by daily inspection and was taken as the first day that the anthers in the middle of the head dehisced. In practice this was when the head was about half extended from the flag leaf. Under these conditions, barley cv Sloop flowers first in the middle two grains and then the flowering spreads up and down the head over several days.

### Antibodies

Rabbit polyclonal anti-peptide antibodies to LTP1 (lab designation V6177) and serpin Z4 (V6175) were produced by Genscript (Piscataway, United States) from antigenic peptides identified within LTP1 (P07597.1; D_33_LHNQAQSSGDRQT_46_) and serpin Z4 (P06293.2; R_258_LSTEPEFIENHIP_271_) respectively, as described in the Supplementary Material of Tanner et al. (2016). Anti-hordein MAbs (lab designation B4 and 23-3) were raised against C-TQQQLQQEQVGQ and C-SFLRPHISQQNS, respectively as in Tanner et al. (2016).

### Total Protein Determination of Grains

Four replicates of two grains were harvested and snap frozen in liquid nitrogen (LN_2_) on the indicated DPA and stored at −80°C until required. Grains were quickly weighed without thawing to determine fresh weight, and then ground in a mortar and pestle under LN_2_, to an ice powder. Either 1 mL (6, 8, 10 DPA) or 2 mL (15, 20, 30, 37 DPA) of extraction buffer containing 8 M urea, 1% (w/v) DTT, 20 mM triethylamine-HCL (termed “Urea/DTT”) and 1/1000 dilution of Sigma plant protease inhibitor (all adjusted to pH 6) was added. The mixture was ground as an ice slurry and allowed to thaw and centrifuged at 15,000 g for 5 min. The supernatants were aliquoted and frozen in LN_2_ and could be thawed and refrozen repeatedly without losing antigenicity or SDS-PAGE performance (Tanner et al., 2013a). Samples were reserved for liquid chromatography tandem mass spectrometry (LC-MS/MS), western blot and ELISA analysis. Total protein was determined by dye-binding (Bradford, 1976).

### Enzyme-Linked Immunosorbent Analysis (ELISA)

Extracts from grains at the indicated DPA were diluted with ELISA Systems sample diluent and added to ELISA wells (ELISA Systems, Brisbane, Australia). ELISA plates were processed according to manufacturers’ instructions. Urea/DTT extracts were diluted 1/1000 with PBST and an appropriate aliquot (50 μL of 6, 8, 10 DPA; or 10 μL of 15, 20, 25, 30, 37 DPA) diluted to 100 μL and calibrated against standard curve of 10–75 ng total hordein extracted from barley cv Sloop and expressed as total hordein (mg/g fresh weight). Total hordein was prepared by adding 20 mg flour into a bead beater to which 20 mg glass beads (0.1 mm, Edwards Instruments, Sydney) plus a stainless steel 10 mm ball bearing in 0.5 mL of 50% (v/v) isopropyl alcohol (IPA), plus 1% (w/v) DTT (IPA/DTT) was added and then extracted for 30 s at a frequency of 1/30 s^−1^, centrifuged at 15,000 g for 5 min, and the process repeated and supernatants pooled. The protein content of the IPA/DTT supernatant was measured (Bradford, 1976) and an aliquot containing 1 mg of hordein was freeze dried and re-dissolved in 1 mL Urea/DTT to yield a 1.0 mg/mL total hordein standard.

### SDS-PAGE

The required aliquot of protein solution was diluted with at least one volume of 6 M urea, 2% (w/v) SDS, 1% (w/v) DTT, 62.5 mM Tris–HCl (pH 6.8), 0.2% (w/v) Bromophenol Blue (termed “Urea/SDS”) at room temperature and loaded on NuPAGE Bis-Tris 4–12% 1 mm gels (Thermo Fisher Scientific) and run at 200 V for 60 min. Protein bands were calibrated against pre-stained protein standards which were in turn calibrated against unstained protein standards (Invitrogen).

### Western Blot

For each primary antibody, duplicate gels were run, with 2 μg of protein per lane, taken from the first and second replicate extractions respectively. Gels were rinsed in distilled water and blotted to nitrocellulose membrane using iBLOT2 (Thermo Fisher Scientific) using 20 V for 1 min; 23 V for 4 min; 25 V for 2 min. The blotted membranes were blocked overnight at 4°C in 1% (w/v) Tween 20, 5% (w/v) Diploma skimmed milk powder in PBST, rinsed and exposed to primary and secondary antibodies as follows. For detection of hordeins, Sigma polyclonal anti-gliadin-HRP was added at a ratio of 1:1000 with incubation for 1 h, washed with PBST for 3 × 10 min and imaged in Amersham ECL reagent. This commercial antibody has previously been shown to detect all gluten and hordein families (Colgrave et al., 2015). For detection of LTP1, anti-LTP (V6177) was added at a ratio of 1:1000 with incubation for 1 h, followed by 3 × 10 min PBST washes, then secondary Amersham donkey anti-rabbit-HRP added at a ratio of 1:1000 with incubation for 1 h, followed by 3 × 10 min PBST washes, and addition of ECL reagent as described above. For serpin detection, anti-serpin Z4 (V6175) was added at a ratio of 1:1000 with incubation for 1 h, followed by 3 × 10 min PBST washes, followed by secondary Amersham donkey anti-rabbit-HRP added at a ratio of 1:1000 with incubation for 1 h, followed by 3 × 10 min PBST washes, and addition of ECL reagent as described above. Representative blots are shown.

### Hordein Mass Spectrometry

#### Protein Digestion

Protein extracts (100 μg protein, *n* = 4) were digested as previously described (Colgrave et al., 2017). In brief, protein was applied to a 10 kDa molecular weight cut-off filter (Millipore, Australia), and washed with two 200 μL of 8 M urea, 100 mM Tris–HCl (pH 8.5) with centrifugation (20,800×*g*, 10 min). For cysteine alkylation, 100 μL of 100 mM iodoacetamide in 8 M urea, 100 mM Tris–HCl was added and incubated at ambient temperature in the dark for 30 min. The filters were centrifuged (20,800×*g*, 10 min) to remove excess iodoacetamide and washed with two 200 μL volumes of 8 M urea, 100 mM Tris–HCl. The buffer was exchanged using 100 mM ammonium bicarbonate (pH 8.0) by two consecutive wash/centrifugation steps. Sequencing grade porcine trypsin or bovine chymotrypsin (Sigma-Aldrich, Australia) at a concentration of 250 μg/mL in 100 mM ammonium bicarbonate with 1 mM CaCl_2_ (200 μL) was added to the protein on the 10 kDa filters and incubated for 16 h at 37°C in a wet chamber. The filters were transferred to fresh centrifuge tubes and the filtrate (digested peptides) were collected by centrifugation (20,800×*g*, 10 min). The filters were washed with 200 μL of 100 mM ammonium bicarbonate and the filtrates were combined and lyophilized. The tryptic peptides were resuspended in 100 μL of 1% formic acid and stored at 4°C until analysis.

#### Global Proteomic Profiling

Gluten-enriched fractions (5 μL; corresponding to 5 μg extracted protein) were analyzed as described previously (Colgrave et al., 2014) with chromatographic separation using a nano HPLC system (Shimadzu Scientific, Rydalmere, Australia) directly coupled to a TripleTOF 5600 MS (SCIEX, Redwood City, CA, United States). ProteinPilot^TM^ 5.0 software (SCIEX) with the Paragon Algorithm (Shilov et al., 2007) was used for protein identification. Tandem mass spectrometry data collected in this study was searched against the Poaceae subset of the Uniprot database (version 2018/08; 1,693,876 sequences). The search parameters were defined as iodoacetamide modified for cysteine alkylation and either trypsin or chymotrypsin as the digestion enzyme. Modifications and cleavages were defined previously (Colgrave et al., 2014). The database search results were manually curated to yield the protein identifications (Supplementary Tables S1, S2) using a 1% global false discovery rate (FDR) determined by the in-built FDR tool within ProteinPilot software (Tang et al., 2008).

#### Identification of Prototypic Peptides

Peptide summaries generated by ProteinPilot (Supplementary File S1) were used to select peptides that yielded intense peaks and were fully tryptic or chymotryptic, i.e., no unusual or missed cleavages. The peptides were subjected to BLAST searching using the Uniprot BLASTp server limited to the taxonomy Poaceae (Supplementary Table S3). MRM transitions were determined for each peptide where the precursor ion (Q1) *m/z* and the fragment ion (Q3) *m/z* values were determined from the data collected in the discovery experiments. The peptides identified in the discovery experiments in this study were added to existing MRM methods as previously described (Colgrave et al., 2016b). Three transitions were used per peptide, with 39 tryptic and 29 chymotryptic peptides measured, wherein the three MRM transitions were required to co-elute and the peak areas of all three were summed (Supplementary Table S4).

#### Targeted MS

Reduced and alkylated tryptic peptides (5 μL, corresponding to 5 μg extracted protein) were chromatographically separated on a Shimadzu Nexera UHPLC and analyzed on a 6500 QTRAP mass spectrometer (SCIEX) as described previously (Colgrave et al., 2014). Quantification was achieved using scheduled MRM scanning experiments using a 40 s detection window for each MRM transition and a 0.3 s cycle time. Peaks were integrated using MultiQuant v3.0 (SCIEX) wherein all three transitions were required to co-elute at the same retention time (RT, min) with a signal-to-noise (S/N) > 3 for detection and a S/N > 5 for quantification. Graphs were generated in Graphpad Prism v6.

#### Phylogenetic Analysis

In total, 22 hordein-like protein sequences identified in the present study were aligned by MUSCLE^1^, and subsequently phylogenetic analysis was performed in MEGA X software (Kumar et al., 2018), using the neighbor-joining method (Saitou and Nei, 1987).

## Results

### Temporal Accumulation of Hordeins

**FIGURE 1:**
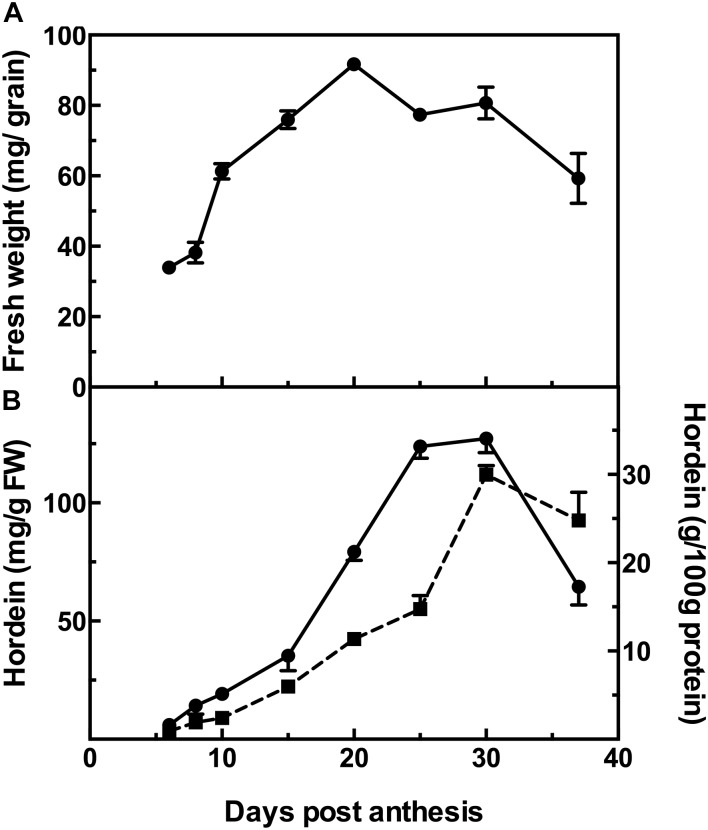
Fresh weight of developing barley grain **(A)**; and hordein content by ELISA **(B)**, calibrated against barley cv Sloop hordein (mg/g fresh weight, FW: 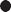, left *y*-axis), or as g/100 g protein (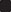, right *y*-axis). Mean (*n* = 4) ± SE are shown; no error bars are shown when SE < symbol size.

The changes during barley grain development can be observed as the increase in the fresh weight of the grains over time, from a low at 6 DPA to a maximum at 20 DPA and then followed by a decrease of 35% at 37 DPA after which the grains are almost entirely desiccated (Figure 1A).

### Hordein Determination by ELISA

The accumulation of hordeins was measured by ELISA, calibrated against an appropriate standard consisting of total hordein purified from barley cv Sloop. Measurement by ELISA shows a steady accumulation in hordein level from near zero levels at 6 DPA to a maximum at 30 DPA. The trends are similar when expressed on either a fresh weight basis or as a proportion of total protein. However, as the seed desiccates beyond this stage, the hordein level as a percentage of total protein decreases by 17% (Figure 1B). The level of hordein on a fresh weight basis decreases more significantly at 37 DPA (by 49%) compared to the peak value observed at 30 DPA (Figure 1B).

### Characterizing the Gluten in the Developing Endosperm

**FIGURE 2:**
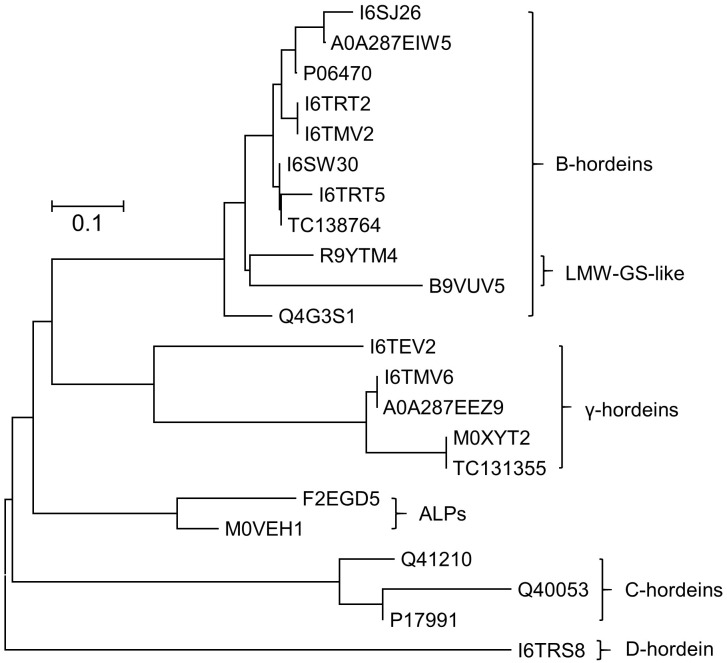
Neighbor-joining phylogenetic tree showing the evolutionary relationship between hordein-like proteins present in *H. vulgare.* All sequences were retrieved from Uniprot database, except two proteins with a “TC” prefix which came from the TIGR database. Sequences were aligned using MUSCLE, and the analyzed in MEGAX (Kumar et al., 2018). The tree is drawn to scale, with branch lengths in the same units as those of the evolutionary distances that were used to infer the phylogenetic tree (scale bar, 0.1 amino acid substitutions per site). Two avenin-like proteins (ALPs) that share homology with the γ-hordeins were identified. Two novel B-hordein isoforms were identified based on peptides that map to wheat low molecular weight glutenins (LMW-GS), demonstrating that despite being absent in the barley genome, they were present in the barley lines studied.

Grains at different stages of development were sampled, frozen and extracted as described above and subsequently subjected to cysteine modification (reduction and alkylation) and digestion using either trypsin or chymotrypsin. The resultant peptide solutions were analyzed by LC-MS/MS and the spectral datasets were searched against the Poaceae subset of the Uniprot-KB database. The protein and peptide summaries were curated to yield the gluten components. The protein identifications in barley cv Sloop (Figure 2) include a suite of B-hordeins, three C-hordeins, a single D-hordein and five γ-hordeins. Additionally, two avenin-like proteins (ALPs: F2EGD5 and M0VEH1) sharing homology with the C-terminal region of the γ-hordeins were identified. These ALPs contain a high proportion of glutamine (Gln; 21–25%) as observed in other gluten families (∼30%), but a lower proportion (∼7%) of proline (Pro) compared to ∼14% present in other hordein families. The ALPs are typically smaller (∼17 kDa) than the γ-hordeins (∼32–33 kDa). Notably, two of the isoforms identified within the B-hordein family included peptides mapping to wheat protein accessions (R9YTM4 and B9VUV5). The issue with protein inference (mapping peptides to proteins) is exacerbated in the absence of complete genomes leading to the identification of orthologous proteins from related species as has occurred here. While the sequence coverage achieved for these protein isoforms was not complete, the peptides identified included amino acid substitutions that were absent in the barley genome. The peptides mapped to a central region of the B-hordeins that is commonly identified by proteomic studies employing trypsin (Colgrave et al., 2017). In total six peptides mapping to this region, starting with the conserved sequence “VFLQQQC,” were identified proving the existence of at least six B-hordeins in cv Sloop. These novel proteins were termed low molecular weight-glutenin-subunit-like B-hordeins (LMW-GS B-hordein). Likewise, one B-hordein (TC138764) and one γ-hordein (TC131355) were identified using translated protein sequences from the TIGR database (Ouyang and Buell, 2004). Since both Golgi and non-Golgi transport routes of hordeins have been proposed the peptide spectral data was investigated for evidence of glycosylation. Both D-hordein and several γ-hordeins contain N-glycosylation consensus sequences (Asn-Xxx-Ser/Thr). Despite the predictions, no evidence of glycosylation was found indicating it was unlikely that the hordeins were transported to the vacuole via the Golgi bodies.

### Western Blot

**FIGURE 3:**
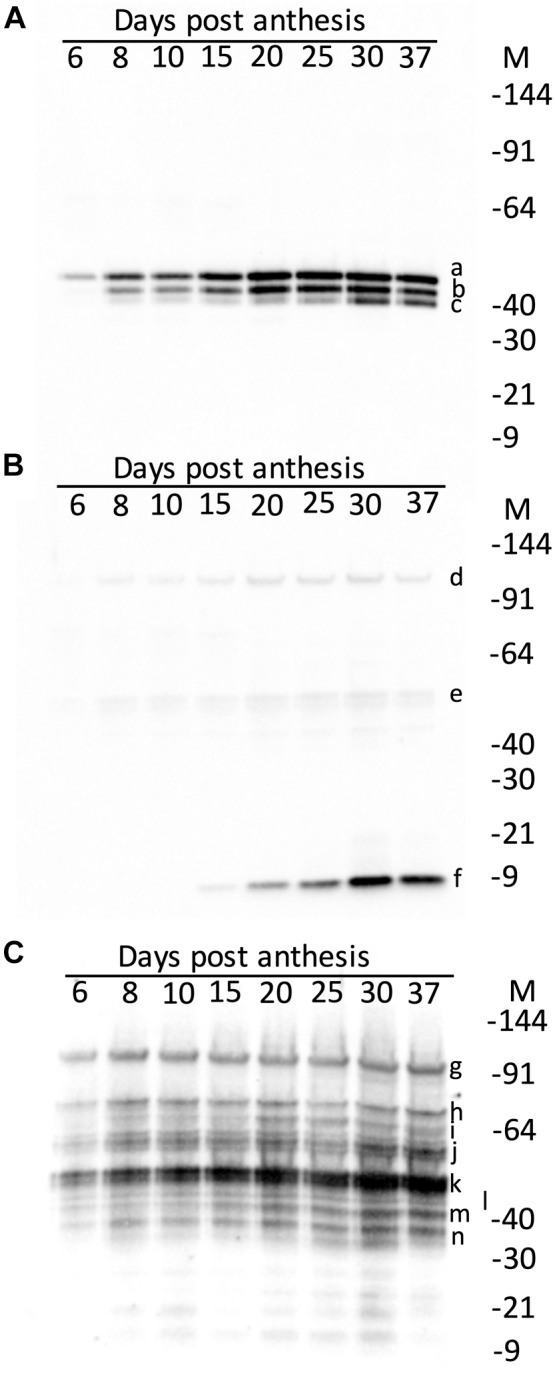
Western blots of developing endosperm showing the effect of maturity (days post-anthesis, DPA) on: **(A)** serpins showing serpin Z7 (**a**, 47.3 kDa), serpin Z4 (**b**, 44.3 kDa) and serpin Z4 minus 4 kDa active loop (**c**, 41.6 kDa); **(B)** LTP1 showing trace artefactual 10-mer (**d**, 97.0 kDa), and 5-mer (**e**, 48.5 kDa) and mature LTP (**f**, 9.7 kDa); and **(C)** hordeins showing D-hordein (**g**, 93.9 kDa), C-hordeins (**h**, 70.5; **i**, 63.7; and **j**, 55.6 kDa), B-hordein (**k**, 47.8 kDa) partly obscuring γ1-hordein (**l**, 45.0 kDa), γ2-hordein (**m**, 40.0 kDa), γ3-hordein (**n**, 38.0 kDa). The relative molecular weights (given in parentheses, in kDa) were determined by calibration against Invitrogen pre-stained standards, which were in turn calibrated against Invitrogen unstained standards.

The effect of maturity on the hordein content of the developing endosperm was firstly examined by western blotting. The accumulation of the abundant grain proteins, the serpins and lipid transfer protein 1 (LTP1), was also studied. These proteins were intended as controls, and in immuno-localisation experiments described in a subsequent publication. The serpins were detected as three isoforms on western blots and the apparent MW calculated relative to protein standards: serpin Z7 (47.3 kDa), native serpin Z4 (44.3 kDa), and as hydrolyzed serpin ZX (minus the 4 kDa active loop, 41.6 kDa) (Roberts et al., 2003). These measured MW correspond to serpin Z7 (Q434392), serpin Z4 (P06293) and serpin ZX (Q40066) with theoretical MW of 42.7, 43.3, and 42.9 kDa, respectively. Serpin Z4 was detectable from 6 DPA, but unlike hordeins the bulk of serpin Z4 accumulation occurred only after mid-development, beyond 15 DPA and reached maximum level at 30 DPA (Figure 3A). Serpin Z4 reaches a maximum at 37 DPA, and does not decrease as some hordeins do, in the later stages of maturity. The remaining control protein LTP1 accumulated late in seed development (Figure 3B) and was not detectable until 15 DPA and accumulated to a maximum at about 30 DPA. Examination of these same proteins using data-dependent LC-MS (data not shown) revealed the same pattern of protein expression with LTP1 (UniProt: P07597) accumulating after 15 days with a 4-fold increase from 25 to 30 DPA. Serpin Z4 (UniProt: P06293) showed a gradual increase from 6 to 30 DPA. Hordeins accumulated early in seed development and were detectable on western blots by 6 DPA (Figure 3C) and the intensity of protein bands increased to a maximum at about 25–30 DPA. The different hordein families, the B-, C-, D-, and γ-hordeins appeared to accumulate synchronously. The ALP, and LMW-GS B-hordein are expected to run on an SDS-PAGE at 17 kDa and 34–40 kDa, respectively, but were not obvious in western blots.

### Mass Spectrometric Analysis

The accumulation of individual hordein isoforms was examined by LC-MRM-MS/MS, using peptide markers representing each of the hordein families (Colgrave et al., 2016b). Most, but not all hordein, prototypic peptides measured by MS rise to a peak at 30 DPA and decreased slightly by 37 DPA. There were some exceptions and it appears that sequence-specific post-translational processing reduced the concentration of some, but not all, isoforms even within the same hordein family.

Several identified proteins do not appear in the barley protein databases but had high amino acid sequence similarity to hordeins (Figure 2). The genetic inter-relationships were revealed by phylogenetic analysis that shows the ALPs are more closely related to the γ-hordeins while the isoforms mapping to wheat LMW-GS proteins were closely related to the barley B-hordein family (Figure 2). These novel proteins generally followed the same trend observed with the other hordeins.

The peptides identified in this study and in previous analyses of barley cv Sloop (Colgrave et al., 2016a) were mapped to the predicted DNA sequences to firstly generate the protein sequence coverage and secondly to confirm that the N-terminal transit peptides were removed from all hordeins (Supplementary Figure S1). Transit peptides were invariably 19 amino acids long for the B-hordeins, ending in amino acids TIA except the two ALPs, which ended with AVA and VQS (Table 1). The C- and D-hordein transit peptides were either 20 or 21 residues long, respectively, ending in TTA. The γ-1-hordein transit peptides were all 19 residues long ending in ATS, while the single γ-3 hordein transit peptide was 15 residues long, ending with ATA. The sequences of three proteins (TC138764, Q40020, and P06471) did not start with MK residues and did not code for transit peptides. It is likely that these sequences represent incomplete protein sequences (protein fragments) rather than processed full-length proteins.

**Table 1:** Hordein transit peptide sequences.

Hordein family	Uniprot accession	Amino acid sequence
ALP	F2EGD5| M0VKM6	MKTMLILALIAFAATSAVA
B1-hordein	I6SJ22	MKTFLIFALLAIAATSTIA
B3-hordein	I6SJ26	MKTFLIFALLAIVATSTIA
LMW-GS-like B-hordein	B9VUV5| R9YTM4	MKTFLVFALLAVAATSAIA
C-hordein	Q41210| Q40053	MKTFLTFVLLAMVMSIVTTA
D-hordein	I6TRS8	MAKRLVLFVAVIVALVALTTA
Gamma-1-hordein	I6TMV6	MKILIILTILAMATTFATS
Gamma-1-hordein	M0XYT2	MKILIILIILAMATSFATS
Gamma-3-hordein	I6TEV2	MKIFLLFSLLGVATA

The protein sequence coverage of hordeins as determined by comparison of LC-MS/MS from this study together with data previously generated, was nearly complete in every case (Supplementary Figure S1) allowing unequivocal identity of hordein isoforms in barley cv Sloop.

**FIGURE 4:**
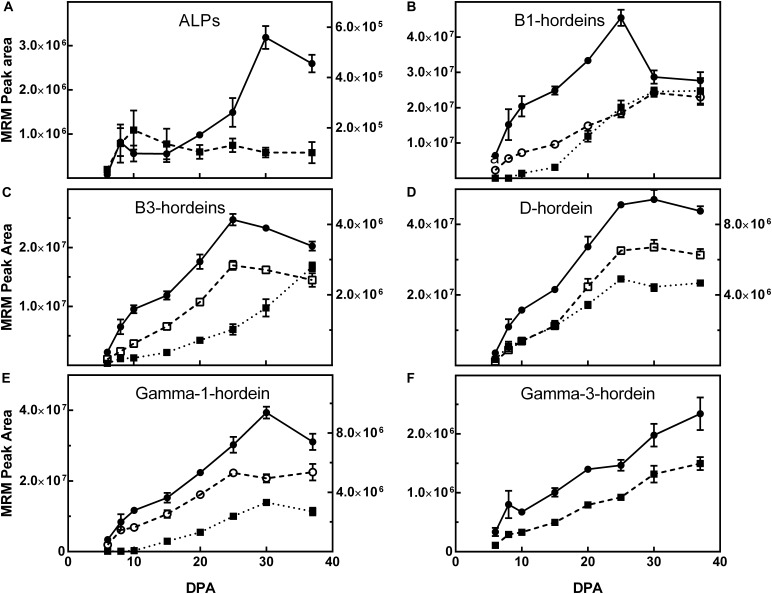
The effect of maturity (days post-anthesis, DPA) on the accumulation of representative hordein family-specific peptides following trypsin cleavage. The mean multiple reaction monitoring (MRM) peak area ± SE (*n* = 3) from 5 μg extracted protein is shown for peptides mapping to: **(A)** avenin-like proteins (ALPs) F2EGD5-T1 (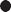, left *y*-axis) and M0VEH1-T1 (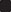, right *y*-axis); **(B)** B1-hordeins I6TRT2-T1 (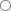),I6TRT2-2 (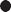), I6TRT2-3 (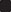), and; **(C)** B3-hordeins Q4G3S1-1 (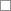, left *y*-axis), Q4G3S1-2 (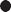, left *y*-axis), and I6SJ26-T1 (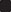, right *y*-axis); **(D)** D-hordein I6TRS8-T1 (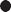, left *y*-axis), I6TRS8-T4 (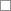, right *y*-axis), and I6TRS8-T5 (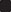, right *y*-axis); **(E)** γ1-hordein I6TMV6-T1 (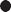, left-*y* axis), TC131355-T1 (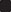, right *y*-axis), I6TMV6-T5 (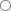, left *y*-axis); and **(F)** γ3-hordein I6TEV2-T1 (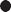), I6TEV2-T2 (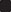). The sequence of proteins and peptide markers are presented in Supplementary Table S4. For clarity, symbols of significance are not shown, but where points differ by 2 × SE, they are significantly different. Detailed statistical comparisons are shown in Supplementary Figures.

**FIGURE 5:**
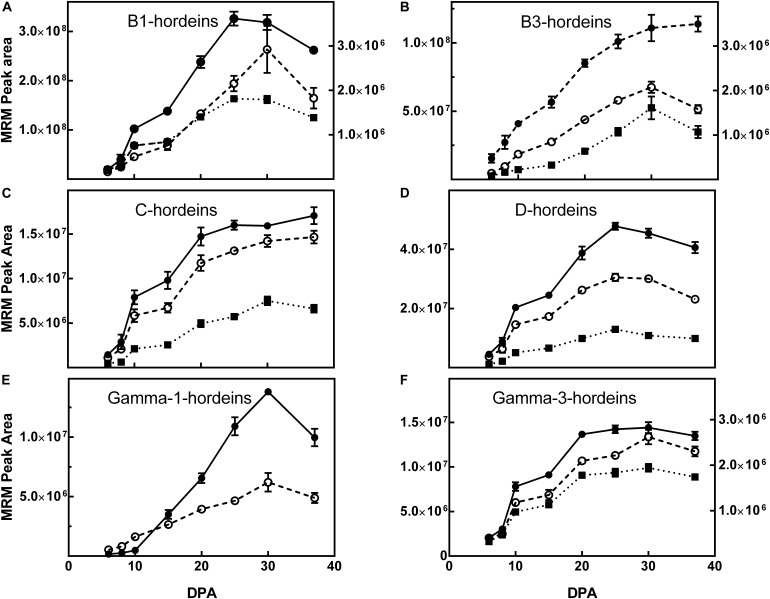
The effect of maturity (days post-anthesis, DPA) on the accumulation of representative hordein family-specific peptides following chymotrypsin cleavage. The mean multiple reaction monitoring (MRM) peak area ± SE (*n* = 3) from 5 μg extracted protein is shown for peptides mapping to: **(A)** B1-hordeins P06470-C1 (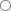, right *y*-axis), P06470-C2 (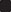, left *y*-axis), and P06470-C3 (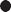, right *y*-axis); **(B)**: B3-hordeins I6SW30-C1 (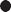, left *y*-axis), I6SJ26-C1 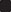, right *y*-axis), I6SJ26-C2 (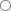, left *y*-axis); **(C)** C-hordeins Q40053-C1 (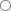), Q40053-C3 (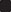) and Q41210-C4 (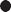); **(D)** D-hordein I6TRS8-C1 (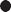), I6TRS8-C5 (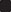) and I6TRS8-C6 (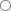); **(E)** γ1-hordein TC131355-C1 (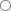), γ1-hordein I6TMV6-C1 (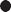); **(F)** γ3-hordein I6TEV2-C1 (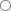, left *y*-axis), γ3 I6TEV2-C2 (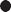, right *y*-axis), and γ3 I6TEV2-C3 (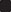, right *y*-axis). The sequence of the proteins and peptide markers are shown in Supplementary Table S4. Where the SE is less than symbol size it is not shown. For clarity, symbols of significance are not shown but where points differ by 2 × SE they are significantly different. Detailed statistical comparisons are shown in Supplementary Figures.

A selection of peptide markers that represented each class of barley gluten were monitored in the grains across the development timeline (DPA). In general, the peptides specific to the different hordein isoforms followed a similar pattern to the total hordein level determined by ELISA, increasing gradually to a maximum at day 30 followed by a slight decrease by 37 DPA. Figure 4 shows the tryptic peptide markers that represent the ALPs, B-hordeins, D-hordein and γ-hordeins. The C-hordeins contain few trypsin cleavage sites and are not well represented after trypsin digestion (Colgrave et al., 2017) and so the accumulation of C-hordeins was followed using chymotryptic peptides (Figure 5).

The D-hordeins increase to a maximum at 37 DPA (Figure 4D) and the avenin-like proteins, B1/B3-hordeins, and γ-1-hordeins increase to a maximum at 25–30 DPA and then decrease by approximately 10% by 37 DPA (Figure 4A,B,C,E). The chymotryptic peptides follow a similar pattern (Figure 5A,B,D,E). However, γ-3-hordein increases gradually to a maximum at 37 DPA and does not decrease when monitoring either tryptic or chymotryptic peptides (Figure 4F, 5F, respectively). Similarly, the C-hordeins accumulate to a maximum at 37 DPA and do not decrease (Figure 5C).

In the tryptic peptide data one of the ALPs (F2EGD5) increased to a significant maximum at 30 DPA (Supplementary Figure S2A), whereas another ALP isoform (M0VEH1) showed low level expression that did not significantly increase after 8 DPA (Supplementary Figure S2B). Generally, most but not all peptides derived from the same protein behave in the same manner. An exception is shown for the B1-hordeins, wherein two peptides, I6TRT2-T1 and -T3, both increased to a maximum value at 37 DPA, whereas the other two peptides in this protein (I6TRT2-T2 and -T4) decreased significantly after 25 DPA (Supplementary Figure S3). The B-hordein peptides monitored are not unique to a single B-hordein isoform (Supplementary Table S3) and as such the different patterns of expression may be the result of cumulatively monitoring more than one protein isoform wherein the isoforms have different protein expression profiles. Good agreement is noted between the two peptides from Q4G3S1 with a non-significant decrease in the level seen beyond 25 DPA (Supplementary Figures S3F,G). Three of the four B3-hordein peptide markers that map to I6SW30 increased to a maximum at 30 DPA with a subtle, but not significant decrease to 37 DPA (Supplementary Figure S4). The remaining peptide (Supplementary Figure S4A) which is notably common to I6SJ26 (I6SJ26-T1) increased to a significant maximum at 37 DPA in agreement with a second peptide mapping to the same isoform (I6SJ26-T2), and the third peptide I6SJ26-T3 decreased beyond 30 DPA. The two peptides mapping to low molecular weight glutenin subunit (LMW-GS: B9VUV5 and R9YTM4) isoforms from wheat, that shared homology with the B-hordeins, show different patterns of protein expression with B9VUV5 reaching a peak at 25 DPA (like B-hordein Q4G3S1) whereas R9YTM4 reached a significant peak at 37 DPA (Supplementary Figure S5).

The six D-hordein peptides all increased reaching a maximum between 25 and 37 DPA (Supplementary Figure S6). The variation noted between 25 and 37 DPA was not significant. The five γ-1-hordein (I6TMV6) peptides all reached a maximum at 30 DPA and decrease slightly to 37 DPA (Supplementary Figure S7). The remaining γ-1-hordein peptides, including the four M0XYT2 peptides and the single TC131355 peptide, increase to a maximum at 37 DPA (Supplementary Figure S8), whereas the single A0A287EEZ9 peptide peaked at 30 DPA. γ -3-Hordein specific peptides I6TEV2-T1 and I6TEV2-T2 both increased to a maximum at 37 DPA (Supplementary Figure S9). All C-hordein specific chymotryptic peptides increased to a significant maximum at either day 30 or 37 DPA (Supplementary Figure S10).

The small, but statistically significant different decreases in protein expression at 37 DPA indicate that sequence-specific differences occur in the accumulation of hordein proteins. Similar patterns are seen in a detailed comparison of the chymotryptic peptides (Figure 5).

## Discussion

It is assumed that extraction of different proteins by a vigorous solvent such as urea/DTT is similar. That many extracted hordeins measured by LC-MS/MS increase to a maximum at 37 DPA and do not decrease confirms that this assumption is correct. This is shown by the minor hordeins, the D-hordeins (Figure 4D), and the γ-3-hordein (Figure 4F). The C-hordeins, represent about 30% of total hordein and also increase to a maximum (Figure 5C). This confirms that the observed decrease in hordein level from 30 to 37 DPA measured by ELISA (Figure 1B) was genuine and not due to a failure to extract protein from the partially desiccated seeds.

Results of ELISA analysis of hordein content, expressed on a protein basis, increased to a maximum at 30 DPA followed by a 17% decrease by 37 DPA. The ELISA Systems kit used the Skerritt antibody, which is selective and detects D- and C-hordeins at least 50× more sensitively than γ- and B-hordeins with half-maximal signals given by 57, 84, 3640, and 19400 ppb hordein, respectively (Tanner et al., 2013a), however, it is unlikely that a change in relative composition is responsible for the decrease observed by ELISA. LC-MS/MS results of 39 tryptic and 29 chymotryptic hordein peptides showed most peptides increased from 6 DPA to a maximum at 30 DPA, often followed by a slight decrease to 37 DPA. ANOVA analysis showed these small decreases were not statistically significant, however, taken together they most likely account for the observed decrease at 37 DPA measured by ELISA.

Some hordeins behave differently compared to other members of the same protein family – either accumulating earlier or decreasing before other family members. This implies fine differential regulation of the expression of hordein genes, and is consistent with a recent study that showed that different classes of hordein transcripts had slightly different expression patterns in developing endosperm (Vinje et al., 2019). Although the reason for this is not apparent it has been observed for protein storage genes in other cereals. For example, a novel family of gliadin genes localized to the wheat group 1 chromosomes (1A, 1B, 1D), and with homology to hordeins was significantly upregulated by nitrogen levels during grain development (Wan et al., 2013). Prolamin gene expression is tissue specific and developmentally regulated, and also sensitive to nitrogen and sulfur nutrition of the grain (Shewry and Halford, 2002).

The biological function of hordeins is unclear, nitrogen storage and involvement in protein trafficking have been suggested (Rechinger et al., 1993; Cameron-Mills et al., 1994). They account for a significant proportion of seed protein, up to 55% (Schalk et al., 2017), and are mobilized during germination. It has been suggested they provide a source of nitrogen for the germinating seed. However, in ULG 2.0, a hordein double null line obtained by combining Risø 56 (no B-hordein) and Risø 1508 (no C-hordeins), the B- and C-hordeins do not accumulate, with only 5% of the total hordein remaining (Tanner et al., 2010) yet this line does not suffer impaired germination compared to wild type cv Sloop (Tanner and Howitt, 2007). Hordeins may play a role in regulating disease resistance as the hordeins have a distant relationship to the amylase inhibitors and serpins of barley, but again ULG 2.0 flour does not preferentially support the growth of bacterial pathogens compared to wild type cv Sloop (Tanner and Howitt, 2007).

The approach used here of analyzing specific proteins by LC-MRM-MS/MS is highly sensitive and selective and hence is applicable to the rapid selection of elite lines. For example, it has been used to produce barley lines carrying significantly reduced hordein content in segregating populations of hordein triple null lines (Tanner et al., 2016). The method may be generalized to the rapid analysis and selection of lines with increased or decreased expression of any protein of agricultural significance.

## Conclusion

Total hordein content detected by ELISA and LC-MS/MS, increased co-ordinately from 6 DPA to a peak level at 30 DPA. Five peptides, members of the B1- and γ1-hordeins decreased significantly by 37 DPA. The majority of hordein peptides including the remaining B1-, B3-, γ-1-, γ-3-, C and D-hordeins increase to a maximum and then either remain high or do not decrease significantly. Hordein accumulation was compared to two other abundant proteins which also accumulate during grain development, LTP1 (a late accumulator, detectable by 15 DPA) and serpin Z4 (an early accumulator, detectable by 6 DPA). In all cases the N-terminal transit peptide, coded for by the hordein genes was not observed in the mature proteins, confirming that these sequences were removed during transit into the RER. Small, but statistically significant, differences in the pattern of accumulation at 37 DPA indicating some sequence-specific differences occur in the accumulation of B-hordein proteins. Similar patterns were observed in a detailed comparison of the protein expression using chymotryptic peptides. Two novel barley B-hordein isoforms were detected mapping to the wheat LMW-GS proteins. From the LC-MS/MS data we can conclude that the pattern of accumulation of these proteins was similar to the bulk of the hordeins. The lack of evidence of hordein N-glycosylation indicated that it was unlikely that the hordeins were transported to the vacuole via the Golgi bodies.

## Author Contributions

GT and MC carried out the experimental work. All authors wrote the manuscript and contributed to the manuscript revision, read and approved the submitted version.

## Conflict of Interest Statement

GT, CH, and MC are authors on patents related to gluten reduction by plant breeding. The remaining authors declare that the research was conducted in the absence of any commercial or financial relationships that could be construed as a potential conflict of interest.

## Footnotes

1 http://www.ebi.ac.uk/Tools/msa/muscle/
