# Supplementary material for: Hordein Accumulation in Developing Barley Grains
Source: Front Plant Sci. 2019 May 16;10:649. doi: 10.3389/fpls.2019.00649 (PMC6532529; doi:10.3389/fpls.2019.00649)
Supplement: Supplementary file 1 [file Data_Sheet_1.pdf]

Supplementary Figures

Avenin-like protein A (F2EGD5):

MKTMLILALIAFAATSAVAQLDTTCSQGYGQCQQQPQQQMNTCAAFLQQCSRTPYVQSQMWQASGCQLMRQQCCQPLAQIS
EQARCQAVCSMAQVIMRQQQGQSFTQPQQQQSQSFGQPQQQVPVEVMRMVLQTLPSMCSVNIPQYCTTTPCSTITPTIYSI
PMAATCAGGVC

B1-hordein (I6SJ22):

MKTFLIFALLAIAATSTIAQQQPLPQQPIPQKPQPYPQQPFPPQQPFPQQPVPQQPQPYPQQPQQPFPPQQPFPQQPPFGL
QQPFPQQPPFGLQQRILSQQQPCTPQQTPLPQGQLYQTLLQLQIPYVHPSILQQLNPCKVFLQQQCSPVRMPQLIARLQML
QLSSCHVLQQQCCQQLPQISEQFRHEAIRAIVYSIFLQEQPQQSVQGVSQTQQQLQQEKVGQCSFQQPQPQQLGQPQQVPQ
SVFLQPHQIAQLEATTSIALRTLPRMCNVNVPLYDIMPPDFWH

B3-hordein (I6SJ26)

MKTFLIFALLAIVATSTIAQQQPYPQQPQPFPQQPIPQQPQPFPQQPQPYPQQPQPFPQQPIPQQPQPYPQQPQPFPQQPI
PQQPQPYPQQPQPFPLQPFPSQQPFPQQPPFWQQQPVLSQQQPCTPQQTPLPQGQQDQMLVQVQIPFVHPSILQQLNPCKV
FLQQQCSPVAMSQRIARSQMLQQSSCYVLQQQCCQQLPQIPEQFRHEAVRAIVYSIVLQEQPQQLVQGVSQPQQQSQLHQV
GQCSFQQPQPQQGQQQQVPQSVFLQPLQLAQLEATASIALRTLPMMCSVNVPFYRILPFGIDTRVGV

B3-hordein (I6TRT5):

MKTFLIFALLAIVATSTIAQQQPYPQQPQPFPQQPQPYPQQPQPFPQQPIPQQPQPYPQQPQPFPLQPFPSQQPFPQQPPF
WQQQPVLSQQQPCTQEQTPLLQEQQDQMLLQVQIPFVHPSILQQLNPCKVFLQQQCSPVAMSQRIARSQMLQQSSCHVLQQ
QCCQQLPQIPEQLRHEAVRAIVYSIVLREQSLQLVQGVSQPQQQSQQQQVGQCSFQQPQPQQGQQQQVPQSVLLQPHQIAQ
LEATTSIALRTLPTMCSVNVPLYRIVPLAIDTRVGV

C-hordein (Q41210):

MKTFLTFVLLAMVMSIVTTARQLNPSSQELQSPQQSYLQQPYPQNPYLPQQPFPVQQPFHTPQQYFPYLPEELSPQYQIPT
PLQPQQPFPQQPQQPLPRPQQPFPWQPQQPFPQPQQPIPYQPQQPFNQQPQQIISQQPQQPFPQQPQQPFPQPQQPFPWQP
QQPFPQPQQPFPLQPQQPFPWQPQQPFPQPQQPIAHQPQQPFSFSQQPQQPFPLQPQQPFPQQPQQPFPQQPQQIIFQQPQ
QSYPVQPQQPFPQPQPVPQQRPQQASPLQPQQPFPQGSEQIIPQQPQQPFPLQP HQPYTQQTIWSMV

C-hordein (Q40053):

MKTFLTFVLLAMVMSIVTTARQLNPSSQELQSPQQSYLQQPYPQNPYLPQKPFPVQQPFHTPQQYFPYLPEELFPQYQIPT
PLQPQQPFPQQPQQPLPRPQQPFPWQPQQPFPQPQEPIPQQPQQPFPQQPQQPFPQQPQQIIFQQPQQSYPVQPQQPFPQP
QPVPQQRPQQASPLQPQPQQASPLQPQQPFPQGSEQIIPQQPFPLQPQPFPQQPQQPLPQPQQPFRQQAELIIPQQPQQPL
PLQPHQPYTQQTIWSMV

D-hordein (I6TRS8):

MAKRLVLFVAVIVALVALTTAEREINGNNIFLDSRSRQLQCERELQESSLEACRRVVDQQLVGQLPWSTGLQMQCCQQLRD
VSPECRPVALSQVVRQYEQQTEVPSKGGSFYPGGTAPPLQQGGWWGTSVKWYYPDQTSSQQSWQGQQGYHQSVTSSQQPGQ
GQQGSYPGSTFPQQPGQGQQPGQRQPWSYPSATFPQQPGQGQGQQGYYPGATSLLQPGQGQQGPYQSATSPQQPGQGQGQQ
ETYPIATSPHQPGQWQQPGQGQQGYYPSVTSPQQSGQGQQGYPSTTSPQQSGQGQQLGQGQQPGQGQQGYPSATFPQQPGQ
WQQGSYPSTTSPQQSGQGQQGYNPSGTSTQQPGQVQQLGQGQQGYYPIATSPQQPGQGQQLGQGQQPGHGQQLVQGQQQGQ
GQQGHYPSMTSPHQTGQGQKGYYPSAISPQQSGQGQQGYQPSGASSQGSVQGACQHSTSSPQQQAQGCQASSPKQGLGSLY
YPSGAYTQQKPGQGYNPGGTSPLHQQGGGFGGGLTTEQPQGGKQPFHCQQTTVSPHQGQQTTVSPHQGQQTTVSPHQGQQT

TVSPHQGQQTTVSPHPGQQTTVSPHQGQQTTVSPHPGQQTTVSPHQGQQTTVSPHQGQQTTVSPHQGQQTTVSPHQGQQTT
VSPHQGQQPGEQPCGFPGQQTTVSLHHGQQSNELYYGSPYHVSVEQPSASLKVAKAQQLAAQLPAMCRLEGGGGLLASQ

Gamma-1-hordein (I6TMV6):

MKILIIILTILAMATTFATS**EMQVNPSVQVQPTQQQPYPESQQPFISQSPQQFPLPQQPFPQQPQQPFPQSQQQCLQQPQHQ**
FPQPTQQFPQRPLLPFTHPFLPFPDQLLPQPPHQSFPQPPQSYPQPPLQPFPQPPQQKYPEQPQQPFPWQQPTIQLYLQQQ
LNSCKEFLLQQCRPVSLLSYLWSKILQQSSCRVMQQQCCLQLAQIPEQYKCTAIDSIVHAIFMQQGQRQGVQIVQQQPQPQ
QVGQCVLVQGQGVAQPQQLAQMEAIRTLVLQSVPSMCNFNVPPNCSTIKAPFVGVVTGVGGQ

Gamma-1-hordein (M0XYT2):

MKILIIILIILAMATSFATS**EMQVNPSVQVQPTQQQTHPESQQPFIHHSQQQFPQPQQSFPQQPQQPFPQ**SQQPCLQQPQHQ
FPQPSQPFPRQPLQPFPR**PFLPFPEQPLPQPPQESFPQPPQSYPQPPLQPFPQPPQESFPQPPQSYPQPLLQPFPQPPQQQ**
YPEQPQQPFPRPPQEQFPNQPQQPFPWQQPSIQLYLQQQLNPCKEFLLQQCRPVSLVSYLWSKIVQQSNCQVMQEQCCLQL
AQIPEQYKCTTIDSIVHAIFMQQGQRQGVQIVQQQPQPQEVGQCVLVQGRDIVQPQ**QLAQMEAIRSLVLQSVPAMCNFNVP**
PNCSTMRAPFFSLVNAGML

Gamma-3-hordein (I6TEV2):

MKIFLLFSLLGVATA**ITTTTMQFNPSGLELERPQQLFPQWQPLPQQPPFLQQEPEQPYPQQQPLPQQQPFPQQPQLPHQHQ**
FPQQLPQQQFPQQMPLQPQQQPQFPQQKPFGQYQQPLTQQPYPQQQPLAQQQPSIEEQHQLNLCKEFLLQQCTLDEKVPLL
QSVISFLRPHISQQNSCQLKRQQCCQQLANINEQSRCPAIQTIVHAIVMQQQQQQVQQQVDHGFVQSQLQQLGQGMPIQLQ
QQPGQAFVLPQQQAQFKVVGSLVIQTLPMLCNVHVPPYCSPFGSMATGSGGQ

Figure S1. Amino acid sequence coverage of representative hordeins from each class as analysed using liquid chromatography mass spectrometry. The discovery data presented here was derived from a previous study of barley cv Sloop (Colgrave *et al*, 2016, Anal. Chem., 88, 9127). Legend: amino-acids in green were confidently identified (≥95%), while amino acids in yellow were identified with 50-95% confidence and those in red with <50% confidence. Residues in grey were not detected. There was no evidence of signal peptides (predicted by SignalP server, underlined).

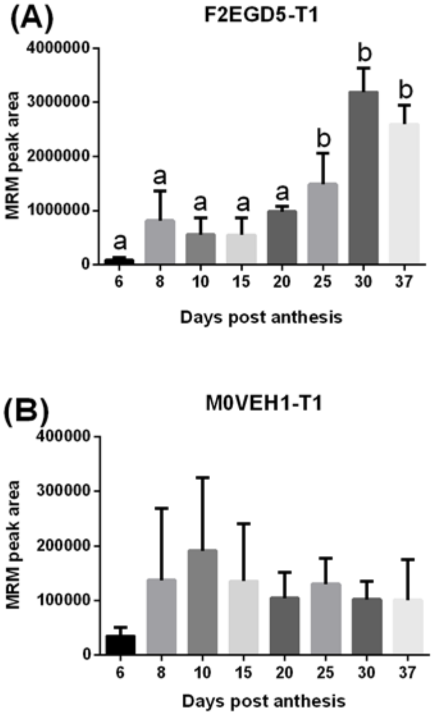

Figure S2. The effect of maturity (days post anthesis, DPA) on the accumulation of the indicated avenin-like protein (ALP) specific peptides following trypsin cleavage. The mean multiple reaction monitoring (MRM) peak area ± SE (n=3) from 5 µg extracted protein is shown for the indicated peptide. Within A, points with different letters were significantly different at $p<0.05$ by one-way ANOVA and Tukey's test. No points were significantly different in B.

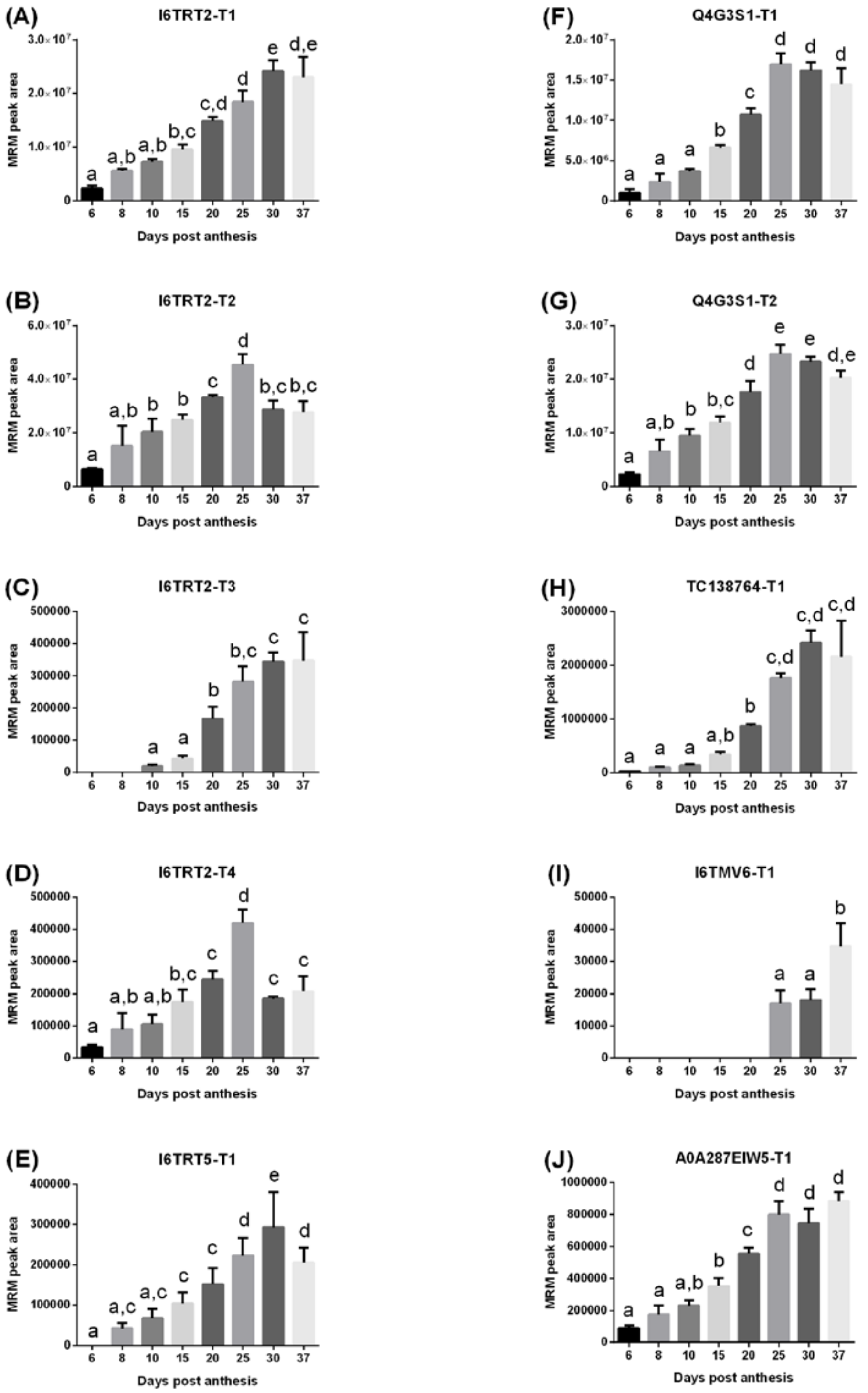

Figure S3. The effect of maturity (days post anthesis, DPA) on the accumulation of the indicated B1-hordein specific peptides following trypsin cleavage. The mean multiple reaction monitoring (MRM) peak area ± SE (n=3) from 5 µg extracted protein is shown for the indicated peptide. Within each graph, points with different letters were significantly different at $p<0.05$ by one-way ANOVA and Tukey's test.

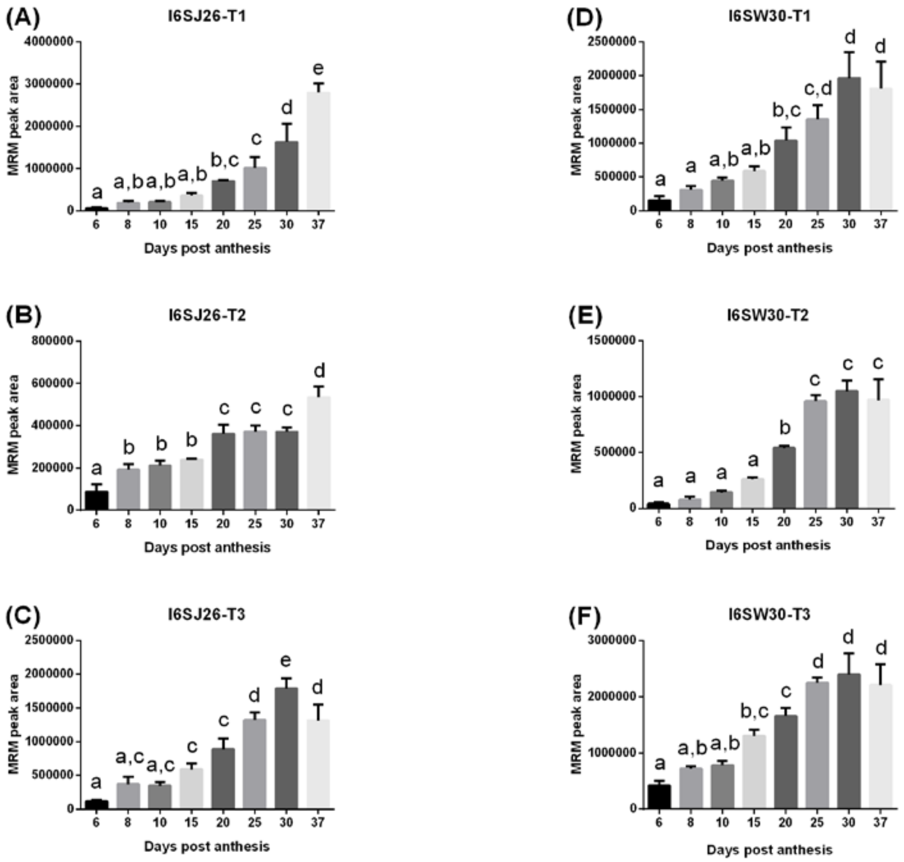

Figure S4. The effect of maturity (days post anthesis, DPA) on the accumulation of B3-hordein specific peptides following trypsin cleavage. The mean multiple reaction monitoring (MRM) peak area ± SE (n=3) from 5 µg extracted protein is shown for the indicated peptide. Within each graph, points with different letters were significantly different at $p<0.05$ by one-way ANOVA and Tukey's test.

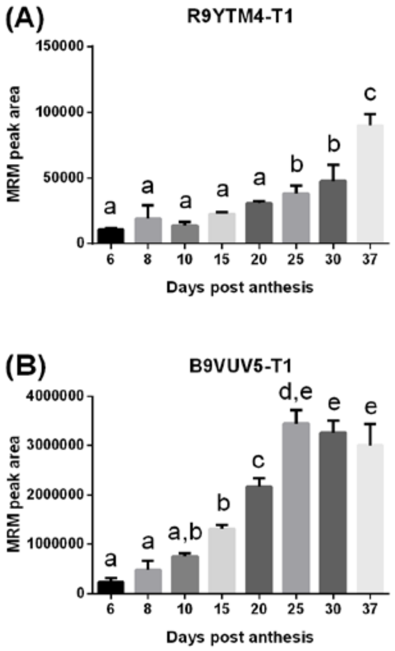

Figure S5. The effect of maturity (days post anthesis, DPA) on the accumulation of the indicated B-hordein (identified by mapping peptides to low molecular weight glutenin subunits, LMW-GS) specific peptides following trypsin cleavage. The mean multiple reaction monitoring (MRM) peak area ± SE (n=3) from 5 µg extracted protein is shown for the indicated peptide. Within each graph, points with different letters were significantly different at $p<0.05$ by one-way ANOVA and Tukey's test.

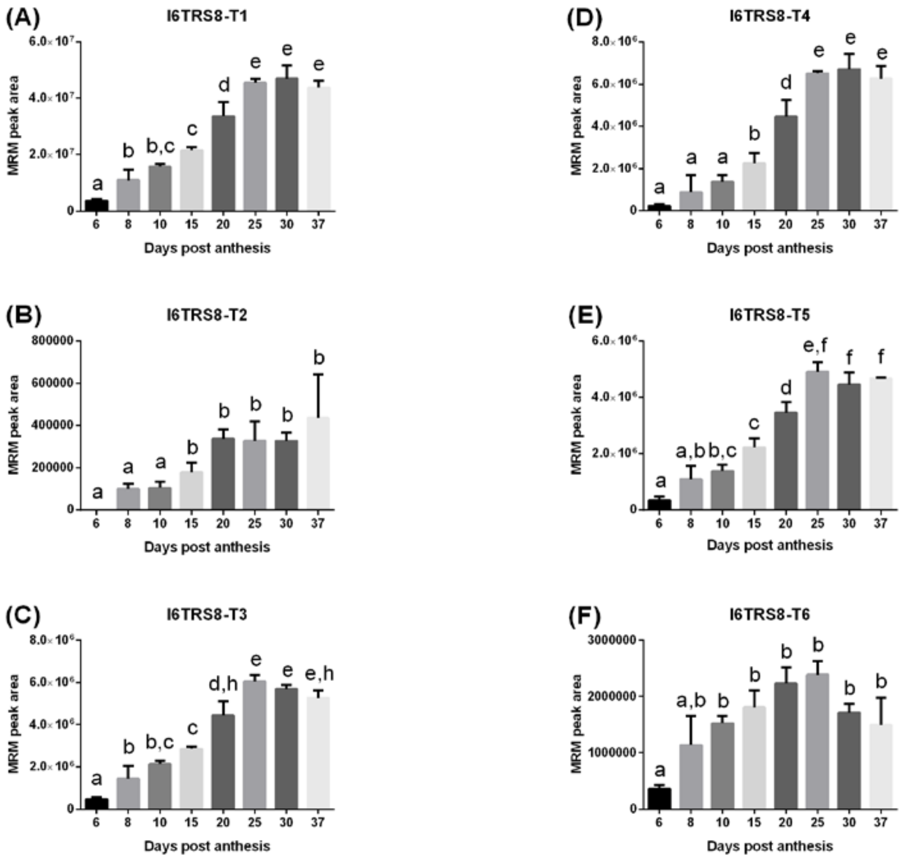

Figure S6. The effect of maturity (days post anthesis, DPA) on the accumulation of the indicated D-hordein specific peptides following trypsin cleavage. The mean multiple reaction monitoring (MRM) peak area ± SE (n=3) from 5 µg extracted protein is shown for the indicated peptide. Within each graph, points with different letters were significantly different at $p<0.05$ by one-way ANOVA and Tukey's test.

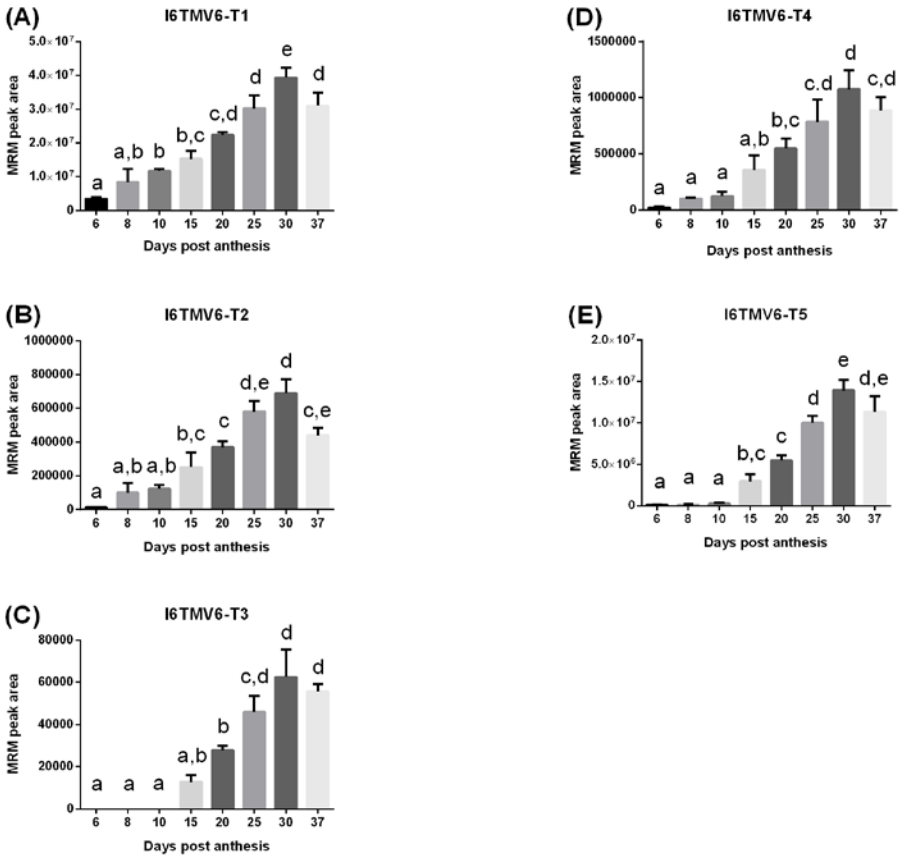

Figure S7. The effect of maturity (days post anthesis, DPA) on the accumulation of the indicated gamma-1-hordein specific peptides following trypsin cleavage. The mean multiple reaction monitoring (MRM) peak area ± SE (n=3) from 5 µg extracted protein is shown for the indicated peptide. Within each graph, points with different letters were significantly different at $p<0.05$ by one-way ANOVA and Tukey’s test.

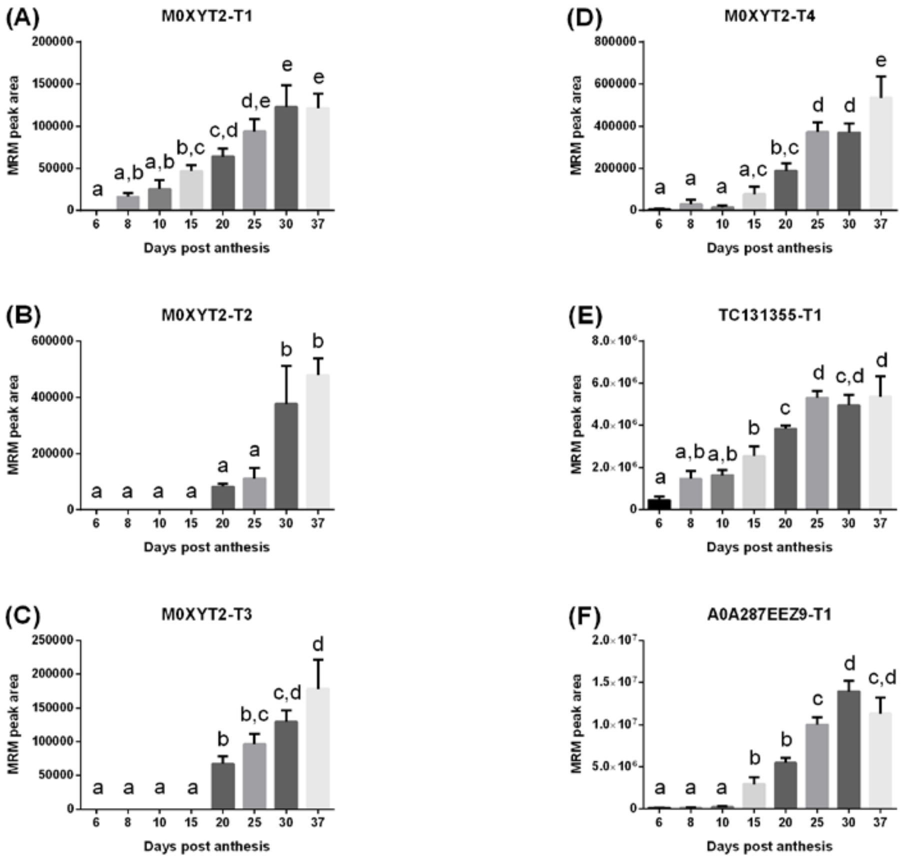

Figure S8. The effect of maturity (days post anthesis, DPA) on the accumulation of the indicated gamma-1-hordein specific peptides following trypsin cleavage. The mean multiple reaction monitoring (MRM) peak area ± SE (n=3) from 5 µg extracted protein is shown for the indicated peptide. Within each graph, points with different letters were significantly different at $p<0.05$ by one-way ANOVA and Tukey's test.

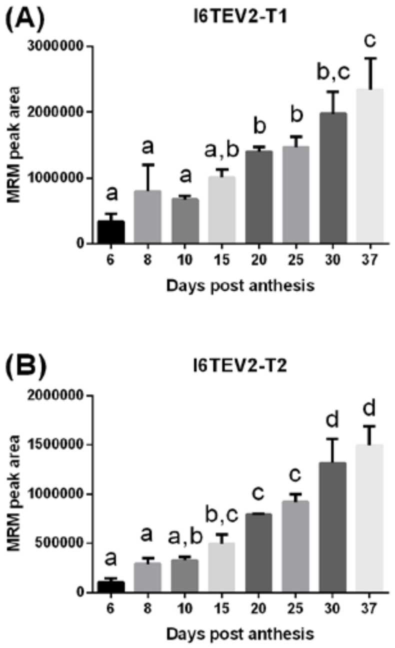

Figure S9. The effect of maturity (days post anthesis, DPA) on the accumulation of the indicated gamma-3-hordein specific peptides following trypsin cleavage. The mean multiple reaction monitoring (MRM) peak area ± SE (n=3) from 5 µg extracted protein is shown for the indicated peptide. Within each graph, points with different letters were significantly different at $p<0.05$ by one-way ANOVA and Tukey's test.

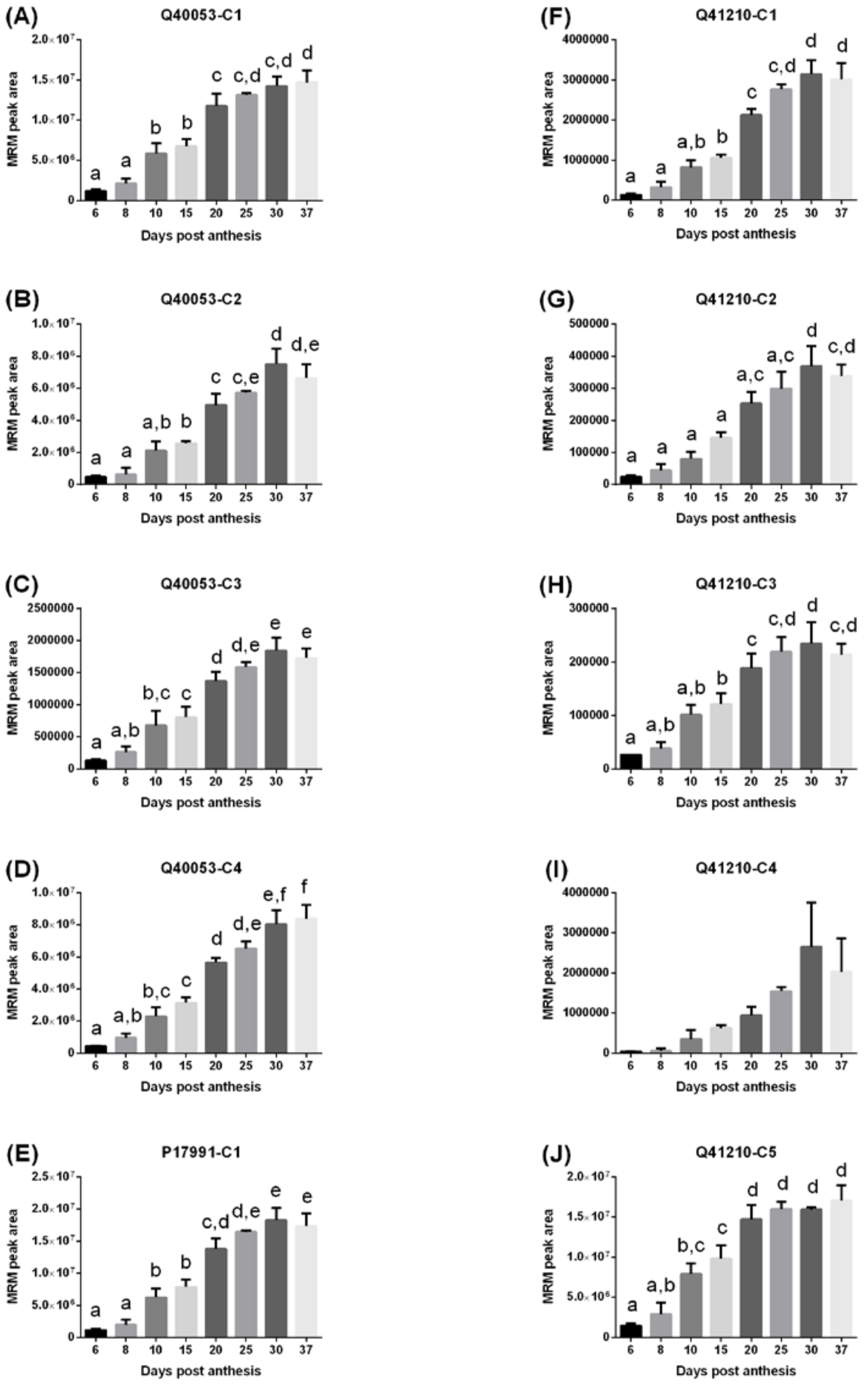

Figure S10. The effect of maturity (days post anthesis, DPA) on the accumulation of the indicated C-hordein specific peptides following chymotrypsin cleavage. The mean multiple reaction monitoring (MRM) peak area ± SE (n=3) from 5 µg extracted protein is shown for the indicated peptide. Within each graph, points with different letters were significantly different at $p<0.05$ by one-way ANOVA and Tukey's test.
