# Supplementary material for: Hordein Accumulation in Developing Barley Grains
Source: Front Plant Sci. 2019 May 16;10:649. doi: 10.3389/fpls.2019.00649 (PMC6532529; doi:10.3389/fpls.2019.00649)
Supplement: Supplementary file 2 [file Data_Sheet_2.pdf]

Table S1. Proteins identified in developing endosperm of barley cv Sloop after trypsin digestion.

N	Unused	Total	% Cov (95)	Accession	Name	Species	Peptides (95%)	Alternate Accession	Alternate Accession	Alternate Accession
1	24.63	24.63	38.10	sp|P06293	Serpin-Z4	*Hordeum vulgare*	22			
2	18.26	18.26	18.21	tr|Q03678	Embryo globulin	*Hordeum vulgare*	16			
**3**	**17.02**	**17.14**	**28.61**	**tr|I6TRS8**	**D-hordein**	***Hordeum vulgare***	**16**			
4	16.21	16.21	32.51	sp|TRY1_B	Trypsin	*Bos taurus*	23			
5	15.04	15.10	21.43	tr|A9Q2Q5	Putative late embryogenesis abundant protein	*Hordeum vulgare*	9			
**6**	**14.87**	**15.02**	**27.67**	**tr|I6TMW0**	**B1-hordein**	***Hordeum vulgare***	**17**	**tr|I6TRT2**	**tr|I6SW25**	**tr|I6SJ22**
7	13.10	13.10	8.67	sp|P31922	Sucrose synthase 1	*Hordeum vulgare*	9			
8	12.89	12.91	15.70	tr|F2D5B2	ATP synthase subunit beta	*Hordeum vulgare*	12			
9	12.30	12.31	26.26	tr|F2CXT7	Fructose-bisphosphate aldolase	*Hordeum vulgare*	13			
10	11.93	11.93	13.03	tr|Q7YMS3	Ribulose bisphosphate carboxylase large chain	*Hordeum chilense*	10			
11	11.88	11.96	23.95	tr|A0A287SR97	Uncharacterized protein (Serpin Z7)	*Hordeum vulgare*	9			
**12**	**11.09**	**11.15**	**27.21**	**tr|I6TMV6**	**Gamma-1-hordein**	***Hordeum vulgare***	**6**			
13	11.05	11.14	44.66	tr|F2EHF8	Predicted protein (Triosephosphate isomerase)	*Hordeum vulgare*	6			
14	10.15	10.16	16.18	sp|P80284	Protein disulfide-isomerase	*Hordeum vulgare*	9			
15	9.62	9.63	11.51	tr|A0A287XP46	Uncharacterized protein (Elongation factor 2)	*Hordeum vulgare*	11			
16	8.66	8.67	10.36	tr|F2CS69	Pyrophosphate--fructose 6-phosphate 1-phosphotransferase subunit beta	*Hordeum vulgare*	4			
17	8.26	8.32	18.32	tr|U5NIU3	Beta-amylase	*Hordeum vulgare*	5			
18	8.26	8.27	7.40	tr|A0A287	Sucrose synthase	*Hordeum vulgare*	5			
19	8.23	8.23	20.05	tr|M0XUU4	Uncharacterized protein (Globulin-1 S allele)	*Hordeum vulgare*	10			
20	8.01	8.01	18.26	tr|A0A287M898	Uncharacterized protein (Thiamine thiazole synthase, chloroplastic)	*Hordeum vulgare*	7			
21	7.78	7.94	17.58	tr|F2CSK4	Predicted protein (Glucose and ribitol dehydrogenase-like protein)	*Hordeum vulgare*	5			
22	7.77	7.91	20.64	tr|M0XLC4	Uncharacterized protein (Adenosine kinase 2)	*Hordeum vulgare*	6			
23	7.16	7.44	15.03	tr|M0USC9	Aspartate aminotransferase	*Hordeum vulgare*	5			
24	6.61	6.77	4.65	tr|F2EBM4	Predicted protein (Vicilin-like antimicrobial peptides 2-2)	*Hordeum vulgare*	6			
25	6.22	6.23	7.18	tr|F2D4A4	Predicted protein (60S ribosomal protein L4)	*Hordeum vulgare*	3			
**26**	**6.14**	**12.22**	**51.45**	**TC138764_F1**	**B1-hordein**	***Hordeum vulgare***	**16**	**tr|Q40022**	**tr|A0A287EFE1**	**sp|P06470**
27	6.12	6.20	17.04	tr|I1H7F9	Uncharacterized protein (Enolase)	*Brachypodium distachyon*	5			
28	6.08	6.16	8.51	tr|F2DCE3	Pyruvate, phosphate dikinase	*Hordeum vulgare*	4			
29	6.01	6.01	4.98	tr|F2EFW5	Predicted protein (Pyrophosphate-energized vacuolar membrane proton pu	*Hordeum vulgare*	4			
30	6.00	6.00	10.58	tr|Q40069	Peroxidase	*Hordeum vulgare*	3			
31	6.00	6.00	13.16	tr|F2EC88	Predicted protein (Rab28 protein)	*Hordeum vulgare*	7			
32	5.51	5.62	12.89	tr|Q4LB20	Adenosylhomocysteinase	*Hordeum vulgare*	4			
33	5.39	5.46	11.11	tr|F2D4W6	Malate dehydrogenase	*Hordeum vulgare*	3			
34	5.29	5.43	18.80	tr|I1IRI1	Uncharacterized protein (60S ribosomal protein L32-1)	*Brachypodium distachyon*	4			
35	5.20	5.27	8.99	tr|U6A1T2	Glucose-1-phosphate adenylyltransferase	*Hordeum vulgare*	3			
36	4.79	4.84	6.14	tr|M0XQ24	Uncharacterized protein (Phosphoglucomutase, cytoplasmic)	*Hordeum vulgare*	4			
37	4.48	4.52	19.18	sp|P16968	Alpha-amylase inhibitor BMAI-1 (Fragment)	*Hordeum vulgare*	6			
38	4.20	4.23	4.54	tr|A0A287V373	Uncharacterized protein (Putative aspartic peptidase A1 family)	*Hordeum vulgare*	2			
39	4.09	4.10	4.66	tr|M0Z2X3	Phosphoglycerate kinase	*Hordeum vulgare*	2			
40	4.07	4.09	4.61	tr|Q40058	HSP70	*Hordeum vulgare*	3			
41	4.05	4.06	9.77	tr|A0A287SBS0	Uncharacterized protein (Late embryogenesis protein D-34-like)	*Hordeum vulgare*	3			
42	4.02	4.02	3.99	tr|F2DCS7	Ketol-acid reductoisomerase	*Hordeum vulgare*	3			
43	4.02	4.02	4.77	sp|P52894	Alanine aminotransferase 2	*Hordeum vulgare*	2			
44	4.00	4.00	7.16	tr|M0XTF9	Uncharacterized protein (T-complex protein 1 subunit alpha)	*Hordeum vulgare*	2			
45	4.01	4.01	6.13	tr|M0YIA7	Isocitrate dehydrogenase [NADP]	*Hordeum vulgare*	2			
46	4.00	18.26	18.21	tr|Q03678	Embryo globulin	*Hordeum vulgare*	16			

**47**	**4.00**	**8.00**	**18.08**	**tr|A0A287EJ06**	**Uncharacterized protein (B-hordein)**	***Hordeum vulgare***	**6**	**tr|A0A287EIZ3 tr|A0A287EIW5**
48	4.00	4.00	7.73	tr|M0X5Z1	Uncharacterized protein (Eukaryotic initiation factor 4A)	*Hordeum vulgare*	2	
49	4.00	4.00	11.87	tr|F2DH85	60S acidic ribosomal protein P0	*Hordeum vulgare*	2	
50	4.00	4.00	7.64	tr|M0XSQ4	Uncharacterized protein (Succinate-semialdehyde dehydrogenase)	*Hordeum vulgare*	2	
51	4.00	4.00	8.39	tr|F2E520	Predicted protein (Uridine 5'-monophosphate synthase)	*Hordeum vulgare*	2	
52	4.00	4.00	19.25	tr|M0UL57	Eukaryotic translation initiation factor 5A	*Hordeum vulgare*	2	
53	4.00	4.00	4.29	sp|P29114	Linoleate 9S-lipoxygenase 1	*Hordeum vulgare*	2	
54	4.00	4.00	11.88	tr|F2EEQ1	Predicted protein	*Hordeum vulgare*	2	
55	4.00	4.00	6.63	tr|F2ECH4	Predicted protein (Late embryogenesis abundant protein)	*Hordeum vulgare*	2	
56	4.00	4.00	8.07	tr|F2DIC8	Predicted protein (Hydroxyproline-rich glycoprotein family protein)	*Hordeum vulgare*	2	
57	4.00	4.00	14.49	tr|F2D4L0	Predicted protein (Glutathione transferase F5)	*Hordeum vulgare*	3	
58	4.00	4.00	11.06	tr|F2CVV8	Superoxide dismutase	*Hordeum vulgare*	2	
59	2.00	2.00	6.55	tr|M0YB08	4-hydroxy-4-methyl-2-oxoglutarate aldolase	*Hordeum vulgare*	1	
60	4.00	4.00	17.01	sp|K1C9_HUMAN	Keratin	*Homo sapiens*	3	
61	3.75	4.03	14.54	tr|F2CUE9	Glyceraldehyde-3-phosphate dehydrogenase	*Hordeum vulgare*	4	
**62**	**3.68**	**3.90**	**22.16**	**tr|M0XYT2**	**Uncharacterized protein (Gamma-1-hordein)**	***Hordeum vulgare***	**3**	
63	3.62	3.77	7.56	tr|F2CQP8	Predicted protein (Lactoylglutathione lyase)	*Hordeum vulgare*	2	
64	3.53	3.68	5.72	tr|M0Z4F0	Uncharacterized protein (Nucleoredoxin)	*Hordeum vulgare*	2	
65	3.47	3.62	3.42	sp|K2C1_HUMAN	Keratin	*Homo sapiens*	2	
66	3.35	3.42	14.71	tr|A0A287H7K0	Uncharacterized protein (Ricin B-like lectin R40G3)	*Hordeum vulgare*	2	
67	3.21	3.31	5.26	tr|F2EGL4	Predicted protein (Eukaryotic peptide chain release factor subunit 1-3)	*Hordeum vulgare*	2	
68	3.17	3.28	8.02	sp|Q43470	14-3-3-like protein B	*Hordeum vulgare*	2	
69	2.99	3.10	9.13	tr|F2E7Q3	Predicted protein	*Hordeum vulgare*	2	
70	2.92	3.06	11.94	tr|V9SHG4	Starch synthase, chloroplastic/amyloplastic	*Hordeum vulgare*	4	
71	2.80	2.89	23.31	tr|F2ELX8	Predicted protein (Chitinase)	*Hordeum vulgare*	4	
72	2.80	2.89	19.05	tr|M0WF11	Uncharacterized protein (Defensin Tm-AMP-D1.2)	*Hordeum vulgare*	4	
73	2.62	2.70	6.81	tr|F2D807	D-3-phosphoglycerate dehydrogenase	*Hordeum vulgare*	2	
74	2.60	2.63	12.84	tr|F2EG53	Predicted protein (Elongation factor 1-beta)	*Hordeum vulgare*	2	
75	2.44	2.47	8.08	tr|F2CUW7	Predicted protein (Universal stress protein A-like protein)	*Hordeum vulgare*	2	
76	2.41	2.53	7.01	tr|F2E9N0	Predicted protein (12S seed storage globulin 1)	*Hordeum vulgare*	3	
77	2.41	2.44	6.60	tr|F2D6B1	Predicted protein (Dehydroascorbate reductase)	*Hordeum vulgare*	1	
78	2.32	2.35	6.22	tr|R7W2E9	ATP synthase subunit alpha	*Aegilops tauschii*	2	
79	2.19	2.21	2.59	tr|A0A287VFE1	Uncharacterized protein (Chaperonin 60-like protein)	*Hordeum vulgare*	1	
80	2.17	2.18	3.64	tr|F2DMV0	Aminopeptidase	*Hordeum vulgare*	2	
81	2.11	2.12	22.22	sp|P07597	Non-specific lipid-transfer protein 1	*Hordeum vulgare*	2	
82	2.11	2.12	12.67	tr|Q96458	17 kDa class I small heat shock protein	*Hordeum vulgare*	1	
83	2.09	2.10	2.34	tr|F2CS40	Aconitate hydratase	*Hordeum vulgare*	1	
84	2.08	5.82	7.34	tr|A0A1W6S456	Ribulose bisphosphate carboxylase large chain	*Nassella trichotoma*	4	
85	2.08	2.09	11.41	tr|Q43769	Oleosin	*Hordeum vulgare*	5	
86	2.03	2.04	2.82	tr|Q6E5A5	Plastidial ADP-glucose transporter	*Hordeum vulgare*	1	
87	2.03	2.03	1.63	tr|M0WX05	Uncharacterized protein (Selenium-binding protein 1)	*Hordeum vulgare*	1	
88	2.02	2.03	6.76	tr|F2EDI8	Predicted protein	*Hordeum vulgare*	1	
89	2.00	2.00	8.40	tr|F2EC14	Predicted protein (60S ribosomal protein L22-2)	*Hordeum vulgare*	1	
90	2.02	2.02	5.78	tr|B5TWK6	rRNA N-glycosidase	*Hordeum vulgare*	1	
91	2.00	7.13	10.04	tr|S4Z8E1	ATP synthase subunit beta, chloroplastic	*Hordeum vulgare*	4	
92	2.01	4.01	3.96	tr|M0YC50	Uncharacterized protein (Cullin-associated NEDD8-dissociated protein 1)	*Hordeum vulgare*	3	
93	2.01	2.02	2.13	tr|F2E4C2	Predicted protein (HSP70)	*Hordeum vulgare*	1	
94	2.01	2.01	20.27	sp|P01086	Trypsin inhibitor CMe	*Hordeum vulgare*	3	
95	2.01	2.01	6.71	tr|A5CFZ1	Tubulin beta chain	*Hordeum vulgare*	3	

96	2.01	2.01	7.90	tr|F2DHH7	Superoxide dismutase [Cu-Zn]	*Hordeum vulgare*	1
97	2.01	2.01	1.75	tr|M0YIZ9	Uncharacterized protein (2-isopropylmalate synthase A)	*Hordeum vulgare*	1
98	2.00	12.91	15.79	tr|F2DJJ2	ATP synthase subunit beta	*Hordeum vulgare*	7
99	2.00	7.91	20.87	tr|A0A1D6AK65	Uncharacterized protein (Adenosine kinase 2)	*Triticum aestivum*	5
100	2.00	6.00	13.46	sp|P82993	Beta-amylase	*Hordeum vulgare*	4
101	2.00	2.06	3.36	tr|Q850M2	Phosphoglycerate kinase (Fragment)	*Hordeum vulgare*	1
102	2.00	4.00	6.10	tr|A0A287GN26	Uncharacterized protein (Aspartic proteinase)	*Hordeum vulgare*	2
103	2.00	4.00	5.20	tr|A0A1Z5R0W2	Uncharacterized protein (NADPH-dependent aldehyde reductase 1)	*Sorghum bicolor*	3
104	2.00	2.44	6.57	tr|A0A2C9PGJ2	Dehydroascorbate reductase 2	*Eleusine coracana*	1
105	2.00	2.12	12.58	tr|F2DGA8	Predicted protein (Small heat shock protein 16.9 kDa)	*Hordeum vulgare*	1
106	2.00	2.04	6.08	tr|A0A287VKP6	Uncharacterized protein (Enolase 1)	*Hordeum vulgare*	1
107	2.00	2.00	1.84	tr|W5GD60	Uncharacterized protein (RuBisCO large subunit-binding protein subunit be	*Triticum aestivum*	1
108	2.00	2.00	2.97	tr|M0XJL4	Uncharacterized protein (Alpha-1,4-glucan-protein synthase)	*Hordeum vulgare*	1
109	2.00	2.00	2.58	tr|M0Z8C6	Predicted protein (Heat shock protein 70)	*Hordeum vulgare*	1
110	2.00	2.00	5.38	tr|F2EL01	Predicted protein (LEA protein)	*Hordeum vulgare*	1
111	2.00	2.00	4.74	tr|A0A287I5P1	Uncharacterized protein (Oxygen-evolving enhancer protein 2)	*Hordeum vulgare*	1
112	2.00	2.00	3.93	tr|M0USG5	Uncharacterized protein (ATP-dependent 6-phosphofructokinase)	*Hordeum vulgare*	1
113	2.00	2.00	3.25	tr|F2D7C4	3-ketoacyl-CoA synthase	*Hordeum vulgare*	1
114	2.00	2.00	2.51	tr|F2CYL7	Predicted protein (Vicilin-like antimicrobial peptides 2-2)	*Hordeum vulgare*	4
115	2.00	2.00	36.00	tr|Q5S741	Chloroplast acetyl-CoA carboxylase (Fragment)	*Hordeum vulgare*	1
116	2.00	2.00	7.43	tr|F2E2L5	Predicted protein (Putative calcium-binding protein CML7)	*Hordeum vulgare*	1
117	2.00	2.00	5.73	tr|F2CQY1	Predicted protein (Putative 40S ribosomal protein)	*Hordeum vulgare*	2
118	2.00	2.00	4.12	sp|P35266	60S ribosomal protein L17-1	*Hordeum vulgare*	1
119	2.00	2.00	9.04	tr|F2DEP3	Predicted protein (60S ribosomal protein L12)	*Hordeum vulgare*	1
120	2.00	2.00	2.99	tr|F2D1P1	Predicted protein (60S ribosomal protein L5)	*Hordeum vulgare*	1
121	2.00	2.00	4.74	tr|F2DHU6	Pyruvate kinase	*Hordeum vulgare*	1
122	2.00	2.00	4.66	tr|F2CRK4	Predicted protein (Enoyl-[acyl-carrier-protein] reductase)	*Hordeum vulgare*	1
123	2.00	2.00	3.30	tr|M0YNH1	Uncharacterized protein (Cinnamyl alcohol dehydrogenase)	*Hordeum vulgare*	1
124	2.00	2.00	1.59	tr|Q9XEI3	Beta-D-glucan exohydrolase isoenzyme ExoI	*Hordeum vulgare*	1
125	2.00	2.00	9.09	tr|M0WDD3	Predicted protein (Triosephosphate isomerase, chloroplastic)	*Hordeum vulgare*	1
126	2.00	2.00	6.78	tr|F2DC21	Predicted protein (Wali7 protein)	*Hordeum vulgare*	1
127	2.00	2.00	21.21	tr|A0A287XZK0	Uncharacterized protein	*Hordeum vulgare*	1
128	2.00	2.00	6.17	tr|F2EI14	Predicted protein (17.5 kDa heat shock protei)	*Hordeum vulgare*	1
129	2.00	2.00	3.93	tr|F2EAD0	Predicted protein (Calcium-dependent lipid-binding (CaLB domain) family p	*Hordeum vulgare*	1
130	2.00	2.00	7.20	tr|O23983	Ascorbate peroxidase	*Hordeum vulgare*	1
131	2.00	2.00	6.64	tr|F2DCF7	Predicted protein (Saposin-like type B, region 1 family protein)	*Hordeum vulgare*	1
132	2.00	2.00	3.68	tr|F2CRB4	Eukaryotic translation initiation factor 3 subunit I	*Hordeum vulgare*	1
133	2.00	2.00	1.28	tr|M0X5Q3	Uncharacterized protein (Carbamoyl-phosphate synthase large chain)	*Hordeum vulgare*	1
134	2.00	2.00	12.96	tr|F2EFB1	Predicted protein	*Hordeum vulgare*	1
135	2.00	2.00	4.81	tr|F2CQQ1	Predicted protein (2-dehydro-3-deoxyphosphooctonate aldolase,)	*Hordeum vulgare*	1
136	2.00	2.00	4.30	tr|A0A287IB81	Eukaryotic translation initiation factor 3 subunit H	*Hordeum vulgare*	1
137	2.00	2.00	2.19	tr|F2E7L1	Histone H4	*Hordeum vulgare*	1
138	2.00	2.00	9.18	tr|A0A287LGK8	Uncharacterized protein (26S protease regulatory subunit 6B)	*Hordeum vulgare*	1
139	2.00	2.00	15.28	tr|O82688	Amino acid selective channel protein	*Hordeum vulgare*	1
140	2.00	2.00	8.85	tr|F2DAK8	Predicted protein (60S acidic ribosomal protein P2A)	*Hordeum vulgare*	1
141	2.00	2.00	15.60	tr|M0WY98	40S ribosomal protein S7	*Hordeum vulgare*	1
142	2.00	2.00	26.53	tr|Q50J89	Putative eukaryotic translation initiation factor (Iso)4E (Fragment)	*Hordeum vulgare*	1
143	2.00	2.00	2.53	tr|M0UL82	Uncharacterized protein (Argininosuccinate synthase)	*Hordeum vulgare*	1
144	2.00	2.00	9.43	tr|B5TWD1	Late embryogenesis abundant protein	*Hordeum vulgare*	1

145	2.00	2.00	12.90	tr|H2E688	Late embryogenesis abundant protein	*Hordeum vulgare*	1
146	2.00	2.00	9.45	tr|A0A287VV58	Uncharacterized protein (40S ribosomal protein S20)	*Hordeum vulgare*	1
147	2.00	2.00	13.58	tr|M0VAW5	Uncharacterized protein (Thioredoxin)	*Hordeum vulgare*	1
148	2.00	2.00	4.58	sp|P29305	14-3-3-like protein A	*Hordeum vulgare*	1
149	2.00	2.00	4.33	tr|M0YTH4	Proline iminopeptidase	*Hordeum vulgare*	1
150	2.00	2.00	5.34	tr|M0UFI4	GTP-binding nuclear protein	*Hordeum vulgare*	1
151	2.00	2.00	2.88	tr|F2D6W5	Ribosomal protein	*Hordeum vulgare*	1
152	2.00	2.00	5.69	tr|F2CPR3	Pyruvate dehydrogenase E1 component subunit beta	*Hordeum vulgare*	1
153	2.00	2.00	6.22	tr|F2D483	40S ribosomal protein S8	*Hordeum vulgare*	1
154	2.00	2.00	2.64	tr|Q94L27	Alcohol dehydrogenase	*Hordeum vulgare*	1
155	2.00	2.00	7.04	tr|Q8S409	Hordoindoline A-1 (Fragment)	*Hordeum vulgare*	1
156	2.00	2.00	1.50	tr|Q8H1Y8	Starch synthase, chloroplastic/amyloplastic	*Hordeum vulgare*	1
157	2.00	2.00	2.19	tr|Q7XZK6	Starch branching enzyme I (Fragment)	*Hordeum vulgare*	1
158	2.00	2.00	4.36	tr|F2DZ70	Aminomethyltransferase	*Hordeum vulgare*	2
159	2.00	2.00	3.55	tr|M0V752	Obg-like ATPase 1	*Hordeum vulgare*	1
160	2.00	2.00	5.66	tr|F2CWL1	Predicted protein (Glutathione-S-transferase)	*Hordeum vulgare*	1
161	2.00	2.00	3.01	tr|Q6BCT3	Methionine synthase	*Hordeum vulgare*	1
162	2.00	2.00	6.37	tr|F2CTC0	Predicted protein (Thioredoxin family Trp26)	*Hordeum vulgare*	1
163	2.00	2.00	3.00	tr|Q4LB12	Methionine synthase 2 enzyme	*Hordeum vulgare*	1
164	2.00	2.00	7.59	tr|Q43770	Oleosin	*Hordeum vulgare*	1
165	2.00	2.00	9.02	tr|F2EG29	Predicted protein (Late embryogenesis abundant protein)	*Hordeum vulgare*	1
166	2.00	2.00	3.27	tr|F2EJY5	4-alpha-glucanotransferase	*Hordeum vulgare*	1
167	2.00	2.00	1.89	tr|F2CY68	Predicted protein (Mitochondrial processing peptidase beta subunit)	*Hordeum vulgare*	1
168	2.00	2.00	1.94	tr|A0A287SNS5	Uncharacterized protein (DEAD-box ATP-dependent RNA helicase 37)	*Hordeum vulgare*	1
169	2.00	2.00	1.80	tr|N1QRM3	Mitochondrial outer membrane porin	*Aegilops tauschii*	1
170	2.00	2.00	2.70	tr|F2CWJ3	Glyceraldehyde-3-phosphate dehydrogenase	*Hordeum vulgare*	1
171	2.00	2.00	3.94	tr|F2E941	Predicted protein (Seryl-tRNA synthetase)	*Hordeum vulgare*	1
172	2.00	2.00	2.15	tr|F2E9B5	Predicted protein	*Hordeum vulgare*	1
173	2.00	2.00	0.63	tr|M0WSS9	Uncharacterized protein (E3 ubiquitin-protein ligase UPL1)	*Hordeum vulgare*	1
174	2.00	2.00	6.15	tr|M8BM34	Uncharacterized protein	*Aegilops tauschii*	1
175	2.00	2.00	6.29	tr|F2DMM2	Predicted protein (Cytochrome c oxidase subunit 5B)	*Hordeum vulgare*	1
176	2.00	2.00	6.91	tr|M0V0Z2	Pyruvate kinase	*Hordeum vulgare*	1
177	2.00	2.00	1.55	tr|M0XYY6	Uncharacterized protein (Clathrin heavy chain)	*Hordeum vulgare*	1
178	2.00	2.00	2.43	tr|F2DL78	Predicted protein (Ubiquitin-like protein)	*Hordeum vulgare*	1
179	2.00	2.00	2.92	tr|M7YC77	Uncharacterized protein	*Triticum urartu*	1
180	2.00	2.00	2.25	tr|M0WUE1	Uncharacterized protein	*Hordeum vulgare*	1
181	2.00	2.00	4.99	tr|M0VXR8	Uncharacterized protein	*Hordeum vulgare*	1
182	2.00	2.00	3.44	tr|F2CRU2	Predicted protein (11-beta-hydroxysteroid dehydrogenase-like protein)	*Hordeum vulgare*	1
183	2.00	2.00	13.04	tr|F2EKY7	Predicted protein	*Hordeum vulgare*	1
184	2.00	2.00	1.61	tr|F2ECB2	Predicted protein (Cytosolic factor-like protein)	*Hordeum vulgare*	1
185	2.00	2.00	4.66	tr|F2E9J4	Predicted protein	*Hordeum vulgare*	1
186	2.00	2.00	2.86	tr|F2E9C4	Predicted protein	*Hordeum vulgare*	1
187	2.00	2.00	7.67	tr|F2E8B4	Predicted protein (Arabinogalactan protein AGP1)	*Hordeum vulgare*	1
188	2.00	2.00	14.13	tr|F2DPX6	Predicted protein	*Hordeum vulgare*	1
189	2.00	2.00	7.03	sp|Q40066	Serpin-ZX	*Hordeum vulgare*	1
190	2.00	2.00	5.79	tr|A1X809	14-3-3D	*Hordeum vulgare*	1
191	2.00	2.00	4.72	tr|F2D692	Predicted protein (Caffeic acid 3-O-methyltransferase)	*Hordeum vulgare*	1
192	2.00	2.00	10.74	sp|P04464	Calmodulin	*Triticum aestivum*	1
193	2.00	2.00	2.76	tr|B9G8U7	Uncharacterized protein (Serine/threonine-protein kinase CTR1)	*Oryza sativa*	1

195	2.00	2.00	16.67	tr|A8V4D2	Chymotrypsin inhibitor-2	*Hordeum vulgare*	1
196	2.00	2.00	4.07	tr|A0A2T7DFZ0	Uncharacterized protein	*9POAL*	1
198	2.00	2.00	5.35	tr|A0A287P882	Uncharacterized protein	*Hordeum vulgare*	1
199	2.00	2.00	3.74	tr|A0A287KCZ7	Uncharacterized protein	*Hordeum vulgare*	1
200	2.00	2.00	29.55	tr|A0A1D6F166	40S ribosomal protein S3-1	*Zea mays*	1
201	2.00	2.00	2.97	tr|A0A1B6Q140	Uncharacterized protein	*Sorghum bicolor*	1
202	2.00	2.00	4.74	tr|A0A0Q3FXQ0	Uncharacterized protein	*Brachipodium distachyon*	1
203	2.00	2.00	16.67	tr|A0A0A9E7A7	Uncharacterized protein	*Arundo dorax*	1
204	2.00	2.00	2.65	sp|K1C15	Keratin	*Ovis aries*	1
205	1.85	2.00	12.34	tr|M0VKU8	6,7-dimethyl-8-ribityllumazine synthase	*Hordeum vulgare*	1
206	1.72	2.00	3.75	tr|F2DFP6	Predicted protein	*Hordeum vulgare*	1
207	1.72	2.00	33.77	tr|A0A1B6P9M1	Uncharacterized protein	*Sorghum bicolor*	1
208	1.68	2.00	4.39	tr|F2D2W7	Predicted protein	*Hordeum vulgare*	1
209	1.68	2.00	2.52	tr|M0XNU3	Uncharacterized protein (HSP70)	*Hordeum vulgare*	1
210	1.68	2.00	5.00	tr|A0A287VI11	Uncharacterized protein	*Hordeum vulgare*	1
211	1.66	2.00	3.49	tr|A0A2S3H2Q0	Uncharacterized protein	*9POAL*	1
212	1.62	2.00	4.46	tr|F2EHX7	Predicted protein	*Hordeum vulgare*	1
213	1.62	2.00	5.93	tr|F2DN06	Predicted protein	*Hordeum vulgare*	1
214	1.46	1.96	3.81	tr|M0Z0Z6	Uncharacterized protein (Auxin-induced protein)	*Hordeum vulgare*	1
215	1.43	1.92	3.21	tr|M0Y7A8	Uncharacterized protein (Phosphoethanolamine methyltransferase)	*Hordeum vulgare*	1
216	1.42	1.92	1.47	tr|A0A1Z5R7G5	Uncharacterized protein	*Sorghum bicolor*	1
217	1.41	1.85	2.39	tr|Q94IC1	Betaine aldehyde dehydrogenase	*Hordeum vulgare*	1
218	1.25	1.72	2.47	tr|F2E6I5	Predicted protein (ATP phosphoribosyltransferase)	*Hordeum vulgare*	1
219	1.24	1.72	5.00	tr|F2CXK6	Predicted protein (3-ketoacyl-CoA thiolase-like protein)	*Hordeum vulgare*	1
220	1.22	1.70	1.69	sp|K1C10	Keratin	*Homo sapiens*	1
221	1.17	1.66	4.63	tr|U5ID99	Maturase K (Fragment)	*9POAL*	1
222	1.14	1.62	4.56	tr|K3XT21	Uncharacterized protein	*Setaria italica*	1
223	1.13	1.60	4.82	tr|F2DM63	Predicted protein (Exonuclease 3'-5' domain-containing protein 1)	*Hordeum vulgare*	1
224	1.13	1.60	5.75	tr|A0A1D6D7T0	Uncharacterized protein	*Triticum aestivum*	1
226	0.99	1.44	2.41	tr|M0W5Z0	Uncharacterized protein (T-complex protein 1 subunit theta)	*Hordeum vulgare*	1
227	0.99	1.44	23.58	tr|A0A1D6MI07	GDSL esterase/lipase	*Zea mays*	1
228	0.98	1.44	5.40	tr|Q8H2M5	Uncharacterized protein	*Oryza sativa*	1
229	0.97	1.42	13.39	tr|M0Y8U3	40S ribosomal protein S12	*Hordeum vulgare*	1
230	0.86	1.30	3.01	tr|F2D3M9	Predicted protein	*Hordeum vulgare*	1
231	0.82	1.26	1.18	tr|K4A546	Uncharacterized protein	*Setaria italica*	1
232	0.78	1.21	3.39	tr|A0A1E5W6Y7	Shugoshin-1	*9POAL*	1
233	0.77	1.22	11.45	tr|A0A1E5UZM9	Tubulin beta chain	*9POAL*	2
234	0.75	1.03	4.98	tr|A0A287VEF2	Uncharacterized protein	*Hordeum vulgare*	1
235	0.74	1.17	4.43	tr|A0A287P659	Uncharacterized protein (27K protein)	*Hordeum vulgare*	1
236	0.64	1.60	3.91	tr|I1UFL8	Maturase K (Fragment)	*9POAL*	1
237	0.61	1.01	1.54	tr|M8BQ64	Putative RING finger protein	*Aegilops tauschii*	1
239	0.52	0.90	16.88	tr|A0A0D3HV86	Histone H2A	*Oryza sativa*	1
240	0.50	0.90	5.20	tr|M0YJN2	Uncharacterized protein (MLP-like protein 34)	*Hordeum vulgare*	1
241	0.47	0.85	3.26	tr|W5EIH4	Uncharacterized protein	*Triticum aestivum*	1
242	0.47	0.85	9.09	tr|A0A0N7KQ06	Os08g0476050 protein (Fragment)	*Oryza sativa*	1
243	0.47	0.84	2.37	sp|Q2QNK7	Solute carrier family 40 member 2, chloroplastic	*ORYSJ*	1
244	0.45	0.82	1.60	tr|W5FHY4	Uncharacterized protein	*Triticum aestivum*	1
245	0.44	2.02	10.98	tr|M0YRV8	Glyceraldehyde-3-phosphate dehydrogenase	*Hordeum vulgare*	3

Table S2. Proteins identified in developing endosperm of barley cv Sloop after chymotrypsin digestion.

N	Unused	Total	% Cov (95)	Accession	Name	Species	Peptides (95%)	Alternate Accession	Alternate Accession
**1**	**35.03**	**35.03**	**63.37**	**tr|A0A287EIM7**	**Uncharacterized protein (C-hordein)**	***Hordeum vulgare***	**67**		
**2**	**18.13**	**18.13**	**25.45**	**tr|I6TRS8**	**D-hordein**	***Hordeum vulgare***	**27**		
3	16.86	16.91	41.63	sp|CTRA	Chymotrypsin	*Bos taurus*	27		
**4**	**13.15**	**13.15**	**34.47**	**tr|A0A287EFE1**	**Uncharacterized protein (B1-hordein)**	***Hordeum vulgare***	**32**	**sp|P06470**	
5	11.78	12.19	19.62	tr|Q7YMR9	Ribulose bisphosphate carboxylase large chain	*Hordeum vulgare*	8		
**6**	**10.49**	**13.16**	**34.96**	**tr|A0A287EEX5**	**Uncharacterized protein (C-hordein)**	***Hordeum vulgare***	**24**		
7	9.37	9.53	22.82	tr|A0A287NA39	Uncharacterized protein (Serpin Z7)	*Hordeum vulgare*	17		
8	8.99	9.33	17.60	tr|F2CXT7	Fructose-bisphosphate aldolase	*Hordeum vulgare*	6		
**9**	**8.64**	**9.37**	**23.93**	**tr|I6TMV6**	**Gamma-1-hordein**	***Hordeum vulgare***	**12**		
10	8.45	8.94	19.30	tr|F2D284	Protein disulfide-isomerase	*Hordeum vulgare*	13		
11	8.39	8.47	10.37	tr|M0XUU4	Uncharacterized protein (Globulin-1 S allele)	*Hordeum vulgare*	8		
12	6.89	6.89	8.37	tr|Q6BCT3	Methionine synthase	*Hordeum vulgare*	5		
13	5.62	5.63	3.68	tr|S4UAT5	Sucrose synthase	*Saccharum officinarum*	3		
14	5.61	5.62	10.09	tr|U5NIU3	Beta-amylase	*Hordeum vulgare*	4		
15	5.39	5.42	7.68	sp|P42210	Phytepsin	*Hordeum vulgare*	3		
16	5.37	5.41	8.39	tr|F2CWX1	Predicted protein (Elongation factor 2)	*Hordeum vulgare*	4		
17	5.36	5.39	7.86	tr|A0A287PR32	40S ribosomal protein SA	*Hordeum vulgare*	3		
18	4.28	4.28	4.40	tr|Q03678	Embryo globulin	*Hordeum vulgare*	2		
19	4.23	4.23	15.12	tr|F2CWR0	Formate dehydrogenase, mitochondrial	*Hordeum vulgare*	4		
20	4.22	4.23	9.62	tr|A0A287U7R7	Glyceraldehyde-3-phosphate dehydrogenase	*Hordeum vulgare*	3		
21	4.13	4.17	14.77	tr|F2D629	Ribulose bisphosphate carboxylase small chain	*Hordeum vulgare*	2		
22	4.08	4.09	10.81	tr|A0A287TT80	Phosphoglycerate kinase	*Hordeum vulgare*	2		
23	4.00	4.00	3.73	tr|Q40025	Beta-glucosidase	*Hordeum vulgare*	2		
24	4.00	4.00	2.77	tr|F2DLM4	Predicted protein (Phospholipase A1-II 7)	*Hordeum vulgare*	2		
25	4.00	4.00	3.86	tr|Q9ZTB6	Starch branching enzyme IIb	*Hordeum vulgare*	2		
26	3.96	4.06	20.69	tr|F2CRF1	Predicted protein (14-3-3 protein)	*Hordeum vulgare*	4		
27	3.69	3.73	3.86	tr|M0X5Z1	Uncharacterized protein (Eukaryotic initiation factor 4A)	*Hordeum vulgare*	3		
28	3.60	3.66	10.41	tr|A0A0U2L8B1	Eukaryotic elongation factor 1 gamma	*Hordeum vulgare*	3		
29	3.37	3.56	5.32	tr|Q42847	Lipoxygenase	*Hordeum vulgare*	4		
30	3.33	3.38	4.72	sp|P11955	26 kDa endochitinase 1	*Hordeum vulgare*	2		
31	3.27	3.35	7.11	tr|F2DCS7	Ketol-acid reductoisomerase	*Hordeum vulgare*	4		
32	3.24	3.30	15.42	tr|F2DD97	Predicted protein (NAP1-related protein 2)	*Hordeum vulgare*	2		
33	3.15	3.22	7.03	tr|Q9MDQ1	ATP synthase subunit alpha (Fragment)	*Sorghum bicolor*	2		
34	3.11	3.19	4.68	tr|F2DCE3	Pyruvate, phosphate dikinase	*Hordeum vulgare*	3		
35	3.10	3.17	6.68	tr|F2D5B2	ATP synthase subunit beta	*Hordeum vulgare*	2		
36	3.06	3.22	5.93	tr|Q4L1B2	Glucose-1-phosphate adenylyltransferase	*Hordeum vulgare*	2		
37	3.05	3.21	14.12	sp|Q43470	14-3-3-like protein B	*Hordeum vulgare*	3		
38	3.02	3.11	12.95	tr|F2EI64	Predicted protein (Late embryogenesis abundant protein)	*Hordeum vulgare*	2		
39	2.96	3.03	23.32	sp|P34937	Triosephosphate isomerase, cytosolic	*Hordeum vulgare*	4		
40	2.90	3.06	5.31	tr|V9SHG9	Starch synthase, chloroplastic/amyloplastic	*Hordeum vulgare*	2		
41	2.88	3.05	6.23	tr|F2D714	Glyceraldehyde-3-phosphate dehydrogenase	*Hordeum vulgare*	2		
42	2.77	2.93	18.47	tr|Q7XUY5	OSJNBb0048E02.12 protein (Major latex protein)	*Oryza sativa*	2		

**43**	**2.74**	**4.98**	**24.15**	**tr|A0A287EFF7**	**Uncharacterized protein (B-hordein)**	***Hordeum vulgare***	**12**			
**44**	**2.73**	**2.80**	**12.88**	**tr|I6TEV2**	**Gamma-3-hordein**	***Hordeum vulgare***	**3**		**tr|A0A287EEZ5**	**sp|P80198**
45	2.64	2.78	2.91	tr|Q6LAA4	Elongation factor 1-alpha	*Hordeum vulgare*	2			
46	2.58	3.60	16.67	tr|A0A287W082	Uncharacterized protein (Protein disulfide-isomerase)	*Hordeum vulgare*	3			
47	2.48	2.48	5.10	tr|M0WQY8	Uncharacterized protein (Heat shock cognate 70 kDa protein 1)	*Hordeum vulgare*	3			
48	2.47	2.62	11.02	sp|P01545	Alpha-hordothionin	*Hordeum vulgare*	2			
49	2.38	2.58	5.11	tr|Q4LB20	Adenosylhomocysteinase	*Hordeum vulgare*	2			
50	2.23	2.25	2.96	tr|M0YIA7	Isocitrate dehydrogenase [NADP]	*Hordeum vulgare*	1			
51	2.16	2.18	3.90	tr|Q40069	Peroxidase	*Hordeum vulgare*	1			
52	2.05	2.05	10.16	tr|Q945R5	Ascorbate peroxidase	*Hordeum vulgare*	3			
53	2.03	2.03	4.68	tr|M0Z903	Pyruvate kinase	*Hordeum vulgare*	1			
54	2.01	2.16	11.79	tr|F2D6B1	Predicted protein (Dehydroascorbate reductase 2)	*Hordeum vulgare*	2			
55	2.01	2.01	18.52	tr|F2E931	40S ribosomal protein S21	*Hordeum vulgare*	2			
56	2.01	2.01	6.87	tr|M0YMF1	Tubulin alpha chain	*Hordeum vulgare*	2			
58	2.00	2.01	6.42	tr|F2EG53	Predicted protein (Elongation factor 1-beta)	*Hordeum vulgare*	1			
59	2.00	2.00	23.89	tr|M0VMI2	Predicted protein (Calmodulin)	*Hordeum vulgare*	2			
60	2.00	2.00	0.85	tr|A0A1D6JEH2	Putative leucine-rich repeat receptor-like protein kinase family protein	*Zea mays*	1			
61	2.00	2.00	2.43	tr|F2D9S0	Predicted protein (Leucine-rich repeat/extensin 2)	*Hordeum vulgare*	1			
62	2.00	2.00	2.09	tr|F2EB61	Pyrophosphate--fructose 6-phosphate 1-phosphotransferase subunit alpha	*Hordeum vulgare*	1			
63	2.00	2.00	16.99	tr|Q945R6	Ascorbate peroxidase (Fragment)	*Hordeum vulgare*	2			
64	2.00	2.00	29.69	tr|A0A287P3F1	Uncharacterized protein (NADH dehydrogenase (Ubiquinone) 1 alpha subcc	*Hordeum vulgare*	1			
65	2.00	2.00	19.67	sp|P69693	Photosystem II reaction center protein K	*Hordeum vulgare*	1			
66	2.00	2.00	5.63	tr|Q9SAT0	NADPH-dependent reductase (Fragment)	*Zea mays*	1			
67	2.00	2.00	7.96	tr|K7R153	Superoxide dismutase [Cu-Zn]	*Hordeum vulgare*	1			
68	2.00	2.00	5.58	tr|Q8W0V1	Cold acclimation WCOR413-like protein gamma form	*Hordeum vulgare*	1			
69	2.00	2.00	1.87	tr|F2DIQ9	Predicted protein (Methylmalonate semialdehyde dehydrogenase)	*Hordeum vulgare*	1			
70	2.00	2.00	2.27	tr|F2D807	D-3-phosphoglycerate dehydrogenase	*Hordeum vulgare*	1			
71	2.00	2.00	3.93	tr|F2DYM4	Predicted protein (Branched-chain amino acid aminotransferase-like)	*Hordeum vulgare*	1			
72	2.00	2.00	2.74	tr|F2DT07	Predicted protein (Plastid phosphate/phosphoenolpyruvate translocator2)	*Hordeum vulgare*	1			
73	2.00	2.00	7.90	tr|M8D9R7	Uncharacterized protein	*Aegilops tauschii*	1			
74	2.00	2.00	7.14	tr|M8BBK0	Uncharacterized protein (Bifunctional epoxide hydrolase 2)	*Aegilops tauschii*	1			
75	2.00	2.00	3.20	tr|M8ABK3	Uncharacterized protein	*Triticum urartu*	1			
76	2.00	2.00	10.00	tr|F2CR57	Predicted protein (Fragment)	*Hordeum vulgare*	1			
77	2.00	2.00	2.82	tr|M0UFT6	Inosine-5'-monophosphate dehydrogenase	*Hordeum vulgare*	1			
78	2.00	2.00	4.76	tr|J3LC80	Uncharacterized protein	*Oryza brachyantha*	1			
79	2.00	2.00	6.72	tr|I1GQ99	Uncharacterized protein (Dof zinc finger protein 7)	*Brachypodium distachyon*	1			
80	2.00	2.00	12.11	tr|F2DYN8	ATP-dependent Clp protease proteolytic subunit	*Hordeum vulgare*	1			
81	2.00	2.00	10.85	tr|F2DVW7	Acyl carrier protein	*Hordeum vulgare*	1			
82	2.00	2.00	4.14	tr|A1C0L3	UCW116, putative lipase	*Hordeum vulgare*	1			
83	2.00	2.00	9.66	sp|P28041	Alpha-amylase/trypsin inhibitor CMa	*Hordeum vulgare*	1			
84	1.92	2.00	9.40	tr|A1X810	14-3-3E	*Hordeum vulgare*	1			
85	1.91	2.00	4.01	tr|F2D4W3	Predicted protein (Enolase)	*Hordeum vulgare*	1			
86	1.85	2.00	14.81	tr|F2E5D0	Predicted protein (Protease)	*Hordeum vulgare*	1			
87	1.72	1.85	4.91	tr|F2DB00	Predicted protein (Prohibitin)	*Hordeum vulgare*	1			
88	1.64	1.86	7.90	tr|F2CQP8	Predicted protein (Lactoylglutathione lyase)	*Hordeum vulgare*	2			

89	1.62	1.74	3.37	tr|A5CFY9	Tubulin beta chain	*Hordeum vulgare*	1
90	1.57	1.70	2.24	tr|Q42835	Beta-D-glucan exohydrolase, isoenzyme ExoII	*Hordeum vulgare*	1
91	1.55	1.68	8.87	sp|P12940	Bowman-Birk type trypsin inhibitor	*Hordeum vulgare*	1
92	1.48	1.60	17.20	tr|A0A287VGJ2	Uncharacterized protein (Acyl-CoA-binding protein)	*Hordeum vulgare*	1
93	1.47	1.59	4.00	tr|Q6E5A5	Plastidial ADP-glucose transporter	*Hordeum vulgare*	1
94	1.43	1.55	5.58	tr|F2DDU3	Predicted protein (20 kDa chaperonin)	*Hordeum vulgare*	1
95	1.43	1.55	10.69	tr|F2E5Q1	Profilin	*Hordeum vulgare*	1
96	1.40	1.51	2.23	tr|F2E0T2	Predicted protein (Leucine aminopeptidase 2)	*Hordeum vulgare*	1
97	1.38	1.49	2.56	tr|S4Z2B0	Photosystem II CP47 reaction center protein	*Hordeum vulgare*	1
98	1.36	1.54	25.36	tr|F2DY31	Predicted protein (Actin-depolymerizing factor 3)	*Hordeum vulgare*	3
99	1.32	1.48	6.48	tr|F2E9N0	Predicted protein (12S seed storage globulin 1)	*Hordeum vulgare*	2

Table S3. Homology searching (using BLASTp analysis) of the barley gluten peptides monitored in this study.

Peptide sequence	Protein type	BLASTp results [a]	Peptide No.	Species
Trypsin-derived peptides				
QQCCQPLAQISEQAR	ALP	**F2EGD5**; A0A287F854; A0A287JK80	F2EGD5-T1	*H. vulgare*
MVLQTLPSMCR	ALP	**M0VEH1**; A0A287JK04; M0VKM6	M0VEH1-T1	*H. vulgare*
VFLQQQCSPVR	B-hordein	A0A0K2GS22; A0A0K2GRU1; A0A0K2GSM3; Q3YAF9; A0A0K1Z5E8; A0A0K2GRS6; A0A287EFB3; A0A287EFH8; A0A287EFF7; I6SJ13; Q40026; I6TMV2; I6QP72; I6SJ22; **I6TRT2**; Q40021; I6R4A7; I6SW25; I6QM99; I6TMW0; Q40020	I6TRT2-T1	*H. vulgare*
MPQLIAR	B-hordein	Q2XQF0; I6SJ13; Q40026; I6TMV2; Q3LTR1; I6QP72; I6SJ22; **I6TRT2**; I6SW25; I6QM99; I6TMW0	I6TRT2-T2	*H. vulgare*
LQMLQLSSCHVLQQQCCQQLPQISEQFR	B-hordein	I6SJ22; **I6TRT2**; I6SW25; I6TMW0	I6TRT2-T3	*H. vulgare*
MCNVNVPLYDIMPPDFWH	B-hordein	A0A287EFB3; Q44026; I6TMV2; I6QP72; I6SJ22; **I6TRT2**; I6SW25; C7FB15; I6QM99; I6TMW0	I6TRT2-T4	*H. vulgare*
VFLQQQCSPVAMSQR	B-hordein	I6TEV5; **I6SJ26**; Q0PIV6; I6SW30; I6TRT5; I6TMW4; A0A287EF93; A0A287EFF5; C7FB14; C7FB17; C7FB17; C7FB13; C7FB16; Q2XQF1; C7FB15; A0A287EF96; A0A287EFB7; A0A287EF74; A0A287EFC4	I6SJ26-T1	*H. vulgare*
TLPMMCSVNVPFYR	B-hordein	**I6SJ26**; Q3YAF9	I6SJ26-T2	*H. vulgare*
ILPFGIDTR	B-hordein	**I6SJ26**; A0A287EIW5; Q3YAF9; A0A287EIZ3; C7FB14; C7FB16; A0A287EF74	I6SJ26-T3	*H. vulgare*
SQMLQQSSCHVLQQQCCQQLPQIPEQLR	B-hordein	P06471; **I6SW30**; I6TRT5; I6TMW4; A0A287EF93; A0A287EFF5; A0A287EF96; A0A287EFB7	I6SW30-T1	*H. vulgare*
TLPTMCSVNVPLYR	B-hordein	P06471; Q9SAT9; I6TEV5; A0A287EFG2; **I6SW30**; I6TRT5; I6TMW4; A0A287EF93; A0A287EFF5; C7FB17; C7FB13; Q2QXQF1; A0A287EF96; A0A287EFB7; A0A287EFB8; A0A287EFB1	I6SW30-T2	*H. vulgare*

IVPLAIDTR	B-hordein	P06471; Q9SAT9; I6TEV5; **I6SW30**; I6TRT5; I6TMW4; A0A287EF93; A0A287EFF5; C7FB13; Q2XQF1; A0A287EF96; A0A287EFB7; A0A287EFB1	I6SW30-T3	*H. vulgare*
AIVYSIVLR	B-hordein	**I6TRT5**	I6TRT5-T1	*H. vulgare*
VFLQQQCSPVPMPQR	B-hordein	Q4GST9; A0A0K2GRS0; A0A287EFG2; A0A0K2GRQ1; Q4G3S6; **Q4G3S1**; Q4G3S5; Q4G3S3; A0A287EFB4; Q4G3S7; Q4G3S8; Q1LZV2; Q4G3T0	Q4G3S1-T1	*H. vulgare*
QLPQIPEQFR	B-hordein	Q4G3S6; **Q4G3S1**; Q4G3S7; Q4G3S8	Q4G3S1-T2	*H. vulgare*
SQLLQQSSCHVLQQQCCQQLPQIPEQFR	B-hordein	**TC138764**	TC138674-T1	*H. vulgare*
LQMLQQSSCHVLQQQCCQQLPQISEQFR	B-hordein	A0A287EFB3; I6SJ13; Q40026; **I6TMV2**; I6QP72; I6QM99	I6TMV2-T1	*H. vulgare*
VFLQQQCSPVAMPQR	B-hordein	**A0A287EIW5**; A0A287EIZ3; A0A287EJ06	A0A287EIW5-T1	*H. vulgare*
VFLQQQCIPVAMSQR	B-hordein	**R9YTM4**	R9YTM4-T1	*T. aestrivum*
VFLQQQCNPVAMPQR	B-hordein	**B9VUV5**	B9VUV5-T1	*T. macha*
ELQESSLEACR	D-hordein	Q84LE9; I6SW23; I6SW34; F2EA67; A0A287G115; **I6TRS8**; Q40054; A0A287FYP1; A0A287FYQ0; A0A287G127; Q40045	I6TRS8-T1	*H. vulgare*
DVSPECRPVALSQVVR	D-hordein	Q84LE9; I6SW23; I6SW34; F2EA67; A0A287G115; **I6TRS8**; Q40054; A0A287FYP1; A0A287FYQ0; A0A287G127; Q40045	I6TRS8-T2	*H. vulgare*
QYEQQTEVPSK	D-hordein	Q84LE9; I6SW23; I6SW34; F2EA67; A0A287G115; **I6TRS8**; Q40054; A0A287FYP1; A0A287FYQ0; A0A287G127; Q40045	I6TRS8-T3	*H. vulgare*
GGSFYPGGTAPPLQQGGWWGTSVK	D-hordein	Q84LE9; I6SW23; I6SW34; F2EA67; A0A287G115; **I6TRS8**; Q40054; A0A287FYP1; A0A287FYQ0; A0A287G127; Q40045	I6TRS8-T4	*H. vulgare*
AQQLAAQLPAMCR	D-hordein	Q84LE9; I6SW23; I6SW34; F2EA67; A0A287G115; **I6TRS8**; Q40054; Q02056	I6TRS8-T5	*H. vulgare*
LEGGGGLLASQ	D-hordein	Q84LE9; I6SW23; I6SW34; F2EA67; A0A287G115; **I6TRS8**; Q40054; Q02056; A0A287G119; A0A287G118	I6TRS8-T6	*H. vulgare*
ILQQSSCR	γ1-hordein	**I6TMV6**; A0A287EEZ9	I6TMV6-T1	*H. vulgare*
VMQQQCCLQLAQIPEQYK	γ1-hordein	P17990; **I6TMV6**; I6SJ17; H8Y0M8; A0A287EEZ9	I6TMV6-T2	*H. vulgare*

CTAIDSIVHAIFMQQGQR	γ1-hordein	P17990; **I6TMV6**; I6SJ17; H8Y0M8; Q70IB4; A0A287EEZ9	I6TMV6-T3	*H. vulgare*
QGVQIVQQQPQPQQVGQCVLVQGQGVAQPQQLAQMEAIR	γ1-hordein	**I6TMV6**	I6TMV6-T4	*H. vulgare*
APFVGVVTGVGGQ	γ1-hordein	P17990; **I6TMV6**; I6SJ17; Q70IB4	I6TMV6-T5	*H. vulgare*
CTTIDSIVHAIFMQQGQR	γ1-hordein	**M0XYT2**	M0XYT2-T1	*H. vulgare*
QGVQIVQQQPQPQEVGQCVLVQGR	γ1-hordein	**M0XYT2**	M0XYT2-T2	*H. vulgare*
DIVQPQQLAQMEAIR	γ1-hordein	**M0XYT2**	M0XYT2-T3	*H. vulgare*
APFFSLVNAGML	γ1-hordein	**M0XYT2**	M0XYT2-T4	*H. vulgare*
DVVQPQQLAQMEAIR	γ1-hordein	**TC131355**	TC131355-T1	*H. vulgare*
APFVGVVSGVGGQ	γ1-hordein	**A0A287EEZ9**	A0A287EEZ9-T1	*H. vulgare*
EFLLQQCTLDEK	γ3-hordein	P80198; A0A287EEZ5; **I6TEV2**	I6TEV2-T1	*H. vulgare*
QQCCQQLANINEQSR	γ3-hordein	P80198; A0A287EEZ5; **I6TEV2**	I6TEV2-T2	*H. vulgare*
Chymotrypsin-derived peptides				
VQGVSQPQQQSQL	B-hordein	**I6SJ26**; A0A287EIW5; A0A287EIZ3; C7FB16; A0A287EJ06	I6SJ26-C1	*H. vulgare*
GIDTRVGV	B-hordein	**I6SJ26**; A0A287EIW4; Q3YAF9; A0A287EIZ3; C7FB14; C7FB16; A0A287EF74	I6SJ26-C2	*H. vulgare*
AIDTRVGV	B-hordein	P06471; Q9SAT9; I6TEV5; **I6SW30**; I6TRT5; I6TMW4; A0A287EF93; A0A287EFF5; C7FB17; C7FB13; Q2XQF1; A0A287EF96; A0A287EFB7; A0A287EFB1	I6SW30-C1	*H. vulgare*
QQEKVGQCSF	B-hordein	I6SJ22; **I6TRT2**; I6SW25; I6TMW0	I6TRT2-C1	*H. vulgare*
GQPQQVPQSVF	B-hordein	A0A0K2GS22; A0A0K2GRU1; A0A0K2GSM3; A0A0K1Z5E8; A0A0K2GRS6; A0A287EFB3; A0A287EFD4; A0A287EFF7; I6SJ13; I6SJ22; **I6TRT2**; Q40021; I6R4A7; I6SW25; I6TMW0; Q40020	I6TRT2-C2	*H. vulgare*
QQKPFPQQPPF	B-hordein	**P06470**; A0A287EFG2; A0A287EFE1; Q3LTR1; Q40022; A0A287EFB4	P06470-C1	*H. vulgare*
RHEAIRAIVY	B-hordein	**P06470**; A0A0K2GS22; A0A0K2GRU1; A0A0K2GSM3; A0A0K2GRS0; Q3YAF9; A0A287EFG2; A0A0K1Z5E8; A0A0K2GRS6; Q2XQF0; A0A287EFB3; A0A287EFD4; A0A287EFH8; A0A287EFF7; I6SJ13; A0A287EFE1; Q40026; I6TMV2; Q3LTR1;	P06470-C2	*H. vulgare*

		I6QP72; I6SJ22; I6TRT2; Q40021; I6R4A7; I6SW25		
RGVGPSVGV	B-hordein	**P06470**; A0A287EFG2; A0A287EFH8; A0A287EFE1; Q0PIV6; Q40022	P06470-C3	*H. vulgare*
LPQKPFPVQQPF	C-hordein	**Q40053**; A0A287EIM7	Q40053-C1	*H. vulgare*
QPQQPFPQGSEQIIPQQPFPL	C-hordein	P06472; A0A287EIM7; I6TEV8; **Q40053**	Q40053-C2	*H. vulgare*
QPQPFPQQPQQPLPQPQQPF	C-hordein	P06472; A0A287EIM7; I6TEV8; **Q40053**	Q40053-C3	*H. vulgare*
IIPQQPQQPLPL	C-hordein	P06472; I6TEV8; **Q40053**	Q40053-C4	*H. vulgare*
LPQQPFPVQQPF	C-hordein	**Q41210**	Q41210-C1	*H. vulgare*
QPQQPFPQQPQQPLPRPQQPFPW	C-hordein	A0A287EIM7; **Q41210**; Q40053	Q41210-C2	*H. vulgare*
QPQQPFPQPQQPIAHQPQQPF	C-hordein	A0A287EIM7; **Q41210**; I6TEV8	Q41210-C3	*H. vulgare*
SQQPQQPFPL	C-hordein	A0A287EIM7; Q40055; **Q41210**; I6TEV8	Q41210-C4	*H. vulgare*
QPQQPFPQQPQQPFPQQPQQIIF	C-hordein	**Q41210**; Q40053	Q41210-C5	*H. vulgare*
IIPQQPQQPFPL	C-hordein	**P17991**	P17991-C1	*H. vulgare*
EQQTEVPSKGGSF	D-hordein	Q84LE9; I6SW23; I6SW34; F2EA67; A0A287G115; **I6TRS8**; Q40054; A0A287FYP1; A0A287FYQ0; A0A287G127; Q40045	I6TRS8-C1	*H. vulgare*
QSATSPQQPGQGQGQQETYPIATSPHQPGQW	D-hordein	I6SW23; I6SW34; F2EA67; **I6TRS8**; Q40054; Q40045	I6TRS8-C2	*H. vulgare*
YPIATSPQQPGQGQQL	D-hordein	Q84LE9; I6SW23; I6SW34; F2EA67; A0A287G115; **I6TRS8**; Q40054; A0A287FYP1; A0A287FYQ0; A0A287G127; Q40045; Q02056	I6TRS8-C3	*H. vulgare*
GQGQQPGHGQQL	D-hordein	Q84LE9; I6SW23; I6SW34; F2EA67; A0A287G115; **I6TRS8**; Q40054; A0A287FYP1; A0A287FYQ0; A0A287G127; Q40045; Q02056	I6TRS8-C4	*H. vulgare*
TQQKPGQGY	D-hordein	Q84LE9; I6SW23; I6SW34; F2EA67; A0A287G115; **I6TRS8**; Q40054; A0A287FYP1; A0A287FYQ0; A0A287G127; Q02056	I6TRS8-C5	*H. vulgare*
HVSVEQPSASL	D-hordein	Q84LE9; I6SW23; I6SW34; F2EA67; A0A287G115; **I6TRS8**; Q40054; Q02056; A0A287G119; A0A287G118	I6TRS8-C6	*H. vulgare*
VGVVTGVGGQ	γ1-hordein	P17990; **I6TMV6**; I6SJ17	I6TMV6-C1	*H. vulgare*
VQGRDVVQPQQL	γ1-hordein	**TC131355**	TC131355-C1	*H. vulgare*
QPQQQPQFPQQKPF	γ1-hordein	P80198; A0A287EEZ5; **I6TEV2**	I6TEV2-C1	*H. vulgare*
AQQQPSIEEQHQL	γ1-hordein	P80198; A0A287EEZ5; **I6TEV2**	I6TEV2-C2	*H. vulgare*

VLPQQQAQF	γ1-hordein	P80198; A0A287EEZ5; **I6TEV2**	I6TEV2-C3	*H. vulgare*

a. Uniprot accession used in paper is shown in bold typefont.

Table S4. Barley gluten peptides monitored in this study. Three MRM transitions were used for each peptide with relative quantitation based on the summed peak area. The protein sequence in the row below each prototypic peptide shows the full protein sequence of the accession; N-terminal sequence (as predicted by SignalP server) identified as missing from mature proteins is italicised. The position of the prototypic peptides are shown in bold.

**Peptide Sequence**	**Peptide marker**	**RT (min)**	**Q1 *m/z* (z)**	**Q3 *m/z* (fragment, z)**	**CE (V)**
QQCCQPLAQISEQAR	F2EGD5-T1	4.32	908.90 (2+)	1112.61 (y10, 1+)<br>1240.67 (y11, 1+)<br>1015.55 (y9, 1+)	45.5
*MKTMLILALIAFAATSAVA*QLDTTCSQGYGQCQQQPQQQMNTCAAFLQQCSRTPYVQSQMWQASGCQLMR**QQCCQPLAQISEQAR**CQAVCSMAQVIMRQQQGQSFTQPQQQQSQSFGQPQQQVPVEVMRMVLQTLPSMCSVNIPQYCTTTPCSTITPTIYSIPMAATCAGGVC					
MVLQTLPSMCR	M0VEH1-T1	5.54	668.32 (2+)	763.34 (y6, 1+)<br>864.39 (y7, 1+)<br>992.44 (y8, 1+)	33.4
*MKTMFIVALIALAVISSVA*QLDTTCIQGYGQCQQQPQHMNTCAAFLQQCSPTPYVQSQMWQASGCQLMRQQCCQPLAQISEQNRCQAVCSVAQVIMQKQQQQQGQSFVQPQQQVPVEITR**MVLQTLPSMCR**VNIPQHCVDTPCSTITQSPYNIPMAATCVGGTC					
VFLQQQCSPVR	I6TRT2-T1	4.17	681.34 (2+)	746.34 (y6, 1+)<br>874.40 (y7, 1+)<br>1002.46 (y8, 1+)	34.1
MPQLIAR	I6TRT2-T2	4.32	414.74 (2+)	472.32 (y4, 1+)<br>600.38 (y5, 1+)<br>697.44 (y6, 1+)	20.7
LQMLQLSSCHVLQQQCCQQLPQISEQFR	I6TRT2-T3	6.70	872.67 (4+)	1004.52 (y8, 1+)<br>1117.60 (y9, 1+)<br>1242.58 (b20, 2+)	31.4
MCNVNVPLYDIMPPDFWH	I6TRT2-T4	8.95	1124.51 (2+)	1157.51 (y9,1+)<br>1320.57 (y10, 1+)<br>1530.71 (y12, 1+)	
*MKTFLIFALLVIAATSTIA*QQQPYPQQPFQPQQPFPQQTIPQQPQPYPQQPQPYPQQPFPPQQEFPQQPPFWPQQPFPQQPPFGLQQRILSQQQPCTPQQTPLPQGQLYQTLLQLQIPYVHPSILQQLNPCK**VFLQQQCSPVRMPQLIARLQMLQLSSCHVLQQQCCQQLPQISEQFR**HEAIRAIVYSIFLQEQPQQSVQGVSQTQQQLQQEKVGQCSFQQPQPQQLGPQQVPQSVFLQPHQIAQLEATTSIALRTLPR**MCNVNVPLYDIMPPDFWH**					
VFLQQQCSPVAMSQR	I6SJ26-T1	4.81	889.93 (2+)	1035.45 (y9, 1+)<br>1163.51 (y10, 1+)<br>1291.57 (y11, 1+)	44.5
TLPMMCSVNVPFYR	I6SJ26-T2	7.14	857.91 (2+)	981.52 (y8, 1+)<br>1141.55 (y9, 1+)<br>1272.59 (y10, 1+)	42.9
ILPFGIDTR	I6SJ26-T3	6.20	516.30 (2+)	561.30 (y5, 1+)<br>708.37 (y6, 1+)	25.8

				805.42 (y7, 1+)	
*MKTFLIFALLAIVATSTIA*QQQPYPQQPQPFPQQPIPQQPQPFPQQPQPYPQQPQPFPQQPIPQQPQPYPQQPQPFPQQPIPQQPQPYPQQPQPFPLQPFPSQQPFPQQPPFWQQQPVLSQQQPCTPQQTPLPQGQQDQMLVQVQIPFVHPSILQQLNPCK**VFLQQQCSPVAMSQR**IARSQMLQQSSCYVLQQQCCQQLPQIPEQFRHEAVRAIVYSIVLQEQPQQLVQGVSQPQQQSQLHQVGQCSFQQPQPQQGQQQQVPQSVFLQPLQLAQLEATASIALR**TLPMMCSVNVPFYRILPFGIDTR**VGV					
SQMLQQSSCHVLQQQCCQQLPQIPEQLR	I6SW30-T1	5.67	863.91 (4+)	980.55 (y8, 1+)<br>1093.64 (y9, 1+)<br>1221.70 (y10, 1+)	31.1
TLPTMCSVNVPLYR	I6SW30-T2	6.25	825.91 (2+)	947.53 (y8, 1+)<br>1107.54 (y9, 1+)<br>1238.58 (y10, 1+)	41.3
IVPLAIDTR	I6SW30-T3	5.26	499.31 (2+)	575.32 (y5, 1+)<br>688.40 (y6, 1+)<br>785.45 (y7, 1+)	25.0
*MKTFLIFALLAIVATSTIA*QQQPYPQQPQPFPQQPIPQQPQPFPQQPQPFPQQPIPQQPQPYPQQPQPFPQQPFPSQQPFPQQPRFWQQQPVLSQQQPCTQEQTPLLQEQQDQMLLQVQIPFVHPSILQQLNPCKVFLQQQCSPVAMSQRIAR**SQMLQQSSCHVLQQQCCQQLPQIPEQLR**HEAVRAIVYSIVLQEQSLQLVQGVSQPQQQSQQQQVGQCSFQQPQPQQGQQQQVPQSVLLQPHQIAQLEATTSIALR**TLPTMCSVNVPLYRIVPLAIDTR**VGV					
AIVYSIVLR	I6TRT5-T1	6.13	517.32 (2+)	587.39 (y5, 1+)<br>750.45 (y6, 1+)<br>849.52 (y7, 1+)	25.9
*MKTFLIFALLAIVATSTIA*QQQPYPQQPQPFPQQPQPYPQQPQPFPQQPIPQQPQPYPQQPQPFPLQPFPSQQPFPQQPPFWQQQPVLSQQQPCTQEQTPLLQEQQDQMLLQVQIPFVHPSILQQLNPCKVFLQQQCSPVAMSQRIARSQMLQQSSCHVLQQQCCQQLPQIPEQLRHEAVR**AIVYSIVLR**EQSLQLVQGVSQPQQQSQQQQVGQCSFQQPQPQQGQQQQVPQSVLLQPHQIAQLEATTSIALRTLPTMCSVNVPLYRIVPLAIDTRVGV					
VFLQQQCSPVPMPQR	Q4G3S1-T1	5.36	907.95 (2+)	628.32 (y5, 1+)<br>1071.49 (y9, 1+)<br>1199.55 (y10, 1+)	45.4
QLPQIPEQFR	Q4G3S1-T2	5.51	628.34 (2+)	676.34 (y5, 1+)<br>789.43 (y6, 1+)<br>1014.54 (y8, 1+)	31.4
*MKTFLIFALLAIAATNTIA*QQQPFPQQPQPYPQQPQPYPQQPFPPQQPFPQQPPFWWQQPVQSQQQPCQQQQTPLPQGQQYQPLLQQQIPFVHPSVLQQLNPCK**VFLQQQCSPVPMPQR**IARSQMLQQSSCHVLQQQCCK**QLPQIPEQFR**HEAIRAIIYSIILQEQQQVQDFVQPQQQQPQQSVQGVSQSQQQSQQPQLGQCSFQQPQLQQLGQQPQQQQVPLWAFLQPQQMAQLEVMTSVALRTLPTMCNVNVPLYGITTSVPLSVGTGVGPY					
SQLLQQSSCHVLQQQCCQQLPQIPEQFR	TC138764-T1	5.95	867.92 (4+)	917.48 (y7, 1+)<br>1014.54 (y8, 1+)<br>1127.62 (y9, 1+)	31.3
MPQRIAR**SQLLQQSSCHVLQQQCCQQLPQIPEQFR**HEAIRAIVYSIFLQEQLQLSVQGVSQPQQQLGQQQVGQCSFQQPQPQQVGQQQQVPQSAFLQPHQIAQLEATTSIALRTLPTMCSVNVPLYRILRGVGPSVGV					
LQMLQQSSCHVLQQQCCQQLPQISEQFR	I6TMV2-T1	5.92	876.42 (4+)	1004.52 (y8, 1+)<br>1117.60 (y9, 1+)<br>1250.07 (b20, 2+)	31.6

*MKTFLIFALLVIAATSTIA*QQQPFPQQPFPQQPQPYPQQPQPYPQQPFQPQQPFPQQTIPQQPQPYPQQPFPPQQAFPQQPPFWPQQPFPQQPPFGLQQPILSQQQPCTPQQTPLPQGQLYQTLLQLQIPYVHPSILQQLNPCKVFLQQQCSPVRMPQLIAR**LQMLQQSSCHVLQQQCCQQLPQISEQFR**HEAIRAIVYSIFLQEQPQQSVQGVSQTQQQLQQEQVGQCSFQQPQPQQLGQAQQVPQSVFLQPHQIAQLEATTSIALRTLPRMCNVNVPLYDIMPPDFWH					
VFLQQQCSPVAMPQR	A0A287EIW5-T1	5.11	894.95 (2+)	1045.47 (y9, 1+)<br>1173.53 (y10, 1+)<br>1301.59 (y11, 1+)	44.8
*MKTFLIFALLAIVATSTIA*QQQPYPQQPQPFPQQPIPQQPQPYPQQPQPFLQQPRPQQPQPYPQQPQPFSQQPIPKQPQPYPQQPQPFPQRPFPSQQPFPQQPPFWQQQPILSKQQPCTPQQPPLPQGQQDQMLVQVQIPFVHPSILQQLNPCK**VFLQQQCSPVAMPQR**IARSQMLQQSSCHVLQQQCCQQLPQIPEQFRHEAVRAIVYSIILQEQPQQLVQGVSQPQQQSQLQQVGQCSFQQPQPQQGKQQQVPQSVFLQPHQLAQLEATASIALRTLPMMCSVNLPLYRILPFGIDTRVGV					
VFLQQQCIPVAMSQR	R9YTM4-T1	4.41	902.95 (2+)	1189.56 (y10, 1+)<br>1317.62 (y11, 1+)<br>1445.68 (y12, 1+)	45.2
*MKTFLIFALLAIAAASAIA*QMETSRVPGLEKPWQQQPLPPQQQPPCSQQQQPFPQQQQPIIILQQSPFSQQQQPVLPQQQPVIILQQPPFSQQQQQQQQQQPFTQQQPPFSQQPPISQQQQQQQQQQQPFTQQQPPFSQQPPISQQQQPPFSQQQQTPFSQQQQIPVIHPSVLQQLNPCK**VFLQQQCIPVAMSQR**LARPQMWQQSSCHVMQQQCCQQLPQIPEQSRSEAIRAIVYSIILQEQQQGFVQPQQQQPQQSGQGVSQHQQQSQQQQQLGQCSFQQPQQLQQLGQQPQQQQIPQGIFLQPHQISQLEVMTSIALCTLPTMCGVNVPLYSSTTIMPFSIGTGVGAY					
VFLQQQCNPVAMPQR	B9VUV5-T1	5.36	908.44 (2+)	912.47 (y8, 1+)<br>1072.48 (y9, 1+)<br>1200.54 (y10, 1+)	45.4
*MKTFLVFALLAVAATSAIA*QMETRCIPGLERPWQQQPLPPQQTFPQQPLFSQQQQQQLFPQQPSFSQQQPPFWQQQPPFSQQQPILPQPPFSQQQQPVLPQQSPFSQQQLVLPPQQQYQQLLQQKVPIVQPSILQQLNPCK**VFLQQQCNPVAMPQR**LARSQMWQQSSCHVMQQQCCQQLPQIPEQFRYEAIRAIIYSIILQEQQQVQGSIQSQQQQPQQLGQCVSQPQQQSQQQLGQQPQQQQLAQGTFLQPHQIAQLEVMTSIALRILPTMCSVNVPLYRTTTSVPFGVGTGVGAY					
ELQESSLEACR	I6TRS8-T1	3.15	661.30 (2+)	735.33 (y6, 1+)<br>822.36 (y7, 1+)<br>951.40 (y8, 1+)	33.1
DVSPECRPVALSQVVR	I6TRS8-T2	4.86	604.65 (3+)	642.85 (y11, 2+)<br>799.42 (y14, 2+)<br>755.90 (y13, 2+)	21.8
QYEQQTEVPSK	I6TRS8-T3	2.32	668.82 (2+)	788.42 (y7, 1+)<br>916.47 (y8, 1+)<br>1045.52 (y9, 1+)	33.4
GGSFYPGGTAPPLQQGGWWGTSVK	I6TRS8-T4	6.90	812. 40 (3+)	920.46 (y8, 3+)<br>977.49 (y8, 3+)<br>1105.54 (y10, 3+)	29.3
AQQLAAQLPAMCR	I6TRS8-T5	4.90	729.36 (2+)	747.34 (y6, 1+)<br>946.44 (y8, 1+)<br>1017.48 (y9, 1+)	36.5
LEGGGGLLASQ	I6TRS8-T6	4.50	501.27 (2+)	584.30 (b7, 1+)<br>697.39 (b8, 1+)<br>768.43 (b9, 1+)	25.1

MAKRLVLFVAVIVALVALTTAEREINGNNIFLDSRSRQLQCER**ELQESSLEACR**RVVDQQLVGQLPWSTGLQMQCCQQLR**DVSPECRPVALSQVVRQYEQQTEVPSKGGSFYPGGT APPLQQGGWWGTSVK**WYYPDQTSSQQSWQGQQGYHQSVTSSQQPGQGQQGSYPGSTFPQQPGQGQQPGQRQPWSYPSATFPQQPGQGQGQQGYYPGATSLLQPGQGQQGPYQSATS PQQPGQGQGQQETYPIATSPHQPGQWQQPGQGQQGYYPSVTSPQQSGQGQQGYPSTTSPQQSGQGQQLGQGQQPGQGQQGYPSATFPQQPGQWQQGSYPSTTSPQQSGQGQQGYNP SGTSTQQPGQVQQLGQGQQGYYPIATSPQQPGQGQQLGQGQQPGHGQQLVQGQQQGQGQQGHYPSMTSPHQTGQGQKGYYPSAISPQQSGQGQQGYQPSGASSQGSVQGACQHSTS SPQQQAQGCQASSPKQGLGSLYYPSGAYTQQKPGQGYNPGGTSPLHQQGGGFGGGLTTEQPQGGKQPFHCQQTTVSPHQGQQTTVSPHQGQQTTVSPHQGQQTTVSPHQGQQTTVS PHPGQQTTVSPHQGQQTTVSPHPGQQTTVSPHQGQQTTVSPHQGQQTTVSPHQGQQTTVSPHQGQQTTVSPHQGQQPGEQPCGFPGQQTTVSLHHGQQSNELYYGSPYHVSVEQPS ASLKVAK**AQQLAAQLPAMCRLEGGGGLLASQ**					
ILQQSSCR	I6TMV6-T1	1.46	496.24 (2+)	509.19 (y4, 1+)<br>637.25 (y5, 1+)<br>765.31 (y6, 1+)	24.8
VMQQQCCLQLAQIPEQYK	I6TMV6-T2	5.97	755.69 (3+)	777.42 (y6, 1+)<br>976.51 (y8, 1+)<br>1089.59 (y9, 1+)	27.2
CTAIDSIVHAIFMQQGQR	I6TMV6-T3	7.82	692.34 (3+)	894.43 (y7, 1+)<br>1007.51 (y8, 1+)<br>1078.55 (y9, 1+)	24.9
QGVQIVQQQPQPQQVGQCVLVQGQGVAQPQQLAQMEAIR	I6TMV6-T4	6.77	1074.56 (4+)	1284.67 (y11, +)<br>1009.56 (b9, 1+)<br>1234.65 (b11, 1+)	38.7
APFVGVVTGVGGQ	I6TMV6-T5	6.04	594.33 (2+)	670.39 (b7, 1+)<br>828.46 (b9, 1+)<br>927.53 (b10, 1+)	29.7
*MKILIIILTILAMATTFATS*EMQVNPSVQVQPTQQQPYPESQQPFISQSPQQFPLPQQPFPQQPQQPFPQSQQQCLQQPQHQFPQPTQQFPQRPLLPFTHPFLPFPDQLLPQPPHQS FPQPPQSYPQPPLQPFPQPPQQKYPEQPQQPFPWQQPTIQLYLQQQLNSCKEFLLQQCRPVSLLSYLWSK**ILQQSSCRVMQQQCCLQLAQIPEQYKCTAIDSIVHAIFMQQGQRQG VQIVQQQPQPQQVGQCVLVQGQGVAQPQQLAQMEAIR**TLVLQSVPSMCNFNVPPNCSTIK**APFVGVVTGVGGQ**					
CTTIDSIVHAIFMQQGQR	M0XYT2-T1	7.80	702.34 (2+)	894.43 (y7, 1+)<br>815.41 (y14, 2+)<br>922.48 (y16, 2+)	25.3
QGVQIVQQQPQPQEVGQCVLVQGR	M0XYT2-T2	5.44	901.80 (3+)	1016.51 (y9, 1+)<br>1115.58 (y10, 1+)<br>1244.62 (y11, 1+)	32.5
DIVQPQQLAQMEAIR	M0XYT2-T3	6.21	870.46 (2+)	931.50 (y8, 1+)<br>1059.56 (y9, 1+)<br>1284.67 (y11, 1+)	43.5
APFFSLVNAGML	M0XYT2-T4	9.17	633.83 (2+)	663.35 (b6, 1+)<br>762.42 (b7, 1+)<br>876.46 (b8, 1+)	31.7
*MKILIILIILAMATSFATS*EMQVNPSVQVQPTQQQTHPESQQPFIHHSQQQFPQPQQSFPQQPQQPFPQSQQPCLQQPQHQFPQPSQPFPRQPLQPFPRPFLPFPEQPLPQPPQES FPQPPQSYPQPPLQPFPQPPQESFPQPPQSYPQPLLQPFPQPPQQQYPEQPQQPFPRPPQEQFPNQPQQPFPWQQPSIQLYLQQQLNPCKEFLLQQCRPVSLVSYLWSKIVQQSNC QVMQEQCCLQLAQIPEQYK**CTTIDSIVHAIFMQQGQRQGVQIVQQQPQPQEVGQCVLVQGRDIVQPQQLAQMEAIR**SLVLQSVPAMCNFNVPPNCSTMR**APFFSLVNAGML**					

DVVQPQQLAQMEAIR	TC131355-T1	5.88	863.45 (2+)	931.50 (y8, 1+)<br>1059.56 (y9, 1+)<br>1284.67 (y11, 1+)	43.2
*MKILIILIILAMATSFATS*EMQVNPSVQVQPTQQQTHPESQQPFIHHSQQQFPQPQQSFPQQPQQPFPQSQQPCLQQPQHQFPQPSQPFPQQPLQSFPRPFLPFPEQPLPQPPQESFPQPPQSYPQPPLQPFPQPPQESFPQPPQSYPQPLLQPFPQPPQQQYPEQPQQPFPRPPQEQFPNQPQQPFPWQQPSIQLYLQQHLNPCKEFLLQQCRPVSLVSYLWSKIVQQSNCQVMQEQCCLQLAQIPEQYKCTTIDSIVHAIFLQQGQRQGVQIVQQQPQPQEVGQCVLVQGR**DVVQPQQLAQMEAIR**SLVLQSVPAMCNFNVPPNCSTMRAPFFSLVNAGML					
APFVGVVSGVGGQ	A0A287EEZ9-T1	5.82	587.32 (2+)	670.39 (b7, 1+)<br>814.45 (b9, 1+)<br>913.52 (b10, 1+)	29.4
*MKILIILTILAMATTFATS*EMQVNPSVQVQPTQQQPYPESQQPFISQSQQQFPQPQQPFPQQPQQPFPQSQQQCLQQPQHXLQQCRPVSLLSYLWSKILQQSSCRVMQQQCCLQLAQIPEQYKCTAIDSIVHAIFMQQGQRQGVQIVQQQPQPQQVGQCVLVQGQGVVQPQQLAQMEAIRTLVLQSVPSMCNFNVPPNCSTIK**APFVGVVSGVGGQ**					
EFLLQQCTLDEK	I6TEV2-T1	5.44	762.36 (2+)	893.38 (y7, 1+)<br>1021.44 (y8, 1+)<br>1134.53 (y9, 1+)	38.1
QQCCQQLANINEQSR	I6TEV2-T2	3.13	938.90 (2+)	1044.54 (y9, 1+)<br>1172.60 (y10, 1+)<br>1300.66 (y11, 1+)	47.0
MKIFLLFSLLGVATAITTTTMQFNPSGLELERPQQLFPQWQPLPQQPPFLQQEPEQPYPQQQPLPQQQPFPQQPQLPHQHQFPQQLPQQQFPQQMPLQPQQQPQFPQQKPFGQYQQPLTQQPYPQQQPLAQQQPSIEEQHQLNLCK**EFLLQQCTLDEK**VPLLQSVISFLRPHISQQNSCQLKR**QQCCQQLANINEQSR**CPAIQTIVHAIVMQQQQQQVQQQVDHGFVQSQLQQLGQGMPIQLQQQPGQAFVLPQQQAQFKVVGSLVIQTLPMLCNVHVPPYCSPFGSMATGSGGQ					
Chymotrypsin-derived peptides					
VQGVSQPQQQSQL	I6SJ26-C1	3.50	713.868	828.421 (y7, 1+)<br>952.485 (b9, 1+)<br>1080.543 (b10, 1+)	34.0
GIDTRVGV	I6SJ26-C2	3.55	408.732	430.277 (y4, 1+)<br>531.325 (y5, 1+)<br>646.352 (y7, 1+)	19.0
*MKTFLIFALLAIVATSTIA*QQQPYPQQPQPFPQQPIPQQPQPFPQQPQPYPQQPQPFPQQPIPQQPQPYPQQPQPFPQQPIPQQPQPYPQQPQPFPLQPFPSQQPFPQQPPFWQQQPVLSQQQPCTPQQTPLPQGQQDQMLVQVQIPFVHPSILQQLNPCKVFLQQQCSPVAMSQRIARSQMLQQSSCYVLQQQCCQQLPQIPEQFRHEAVRAIVYSIVLQEQPQQL**VQGVSQPQQQSQL**HQVGQCSFQQPQPQQGQQQQVPQSVFLQPLQLAQLEATASIALRTLPMMCSVNVPFYRILPF**GIDTRVGV**					
AIDTRVGV	I6SW30-C1	3.50	415.740	430.277 (y4, 1+)<br>531.325 (y5, 1+)<br>646.352 (y6, 1+)	19.4
*MKTFLIFALLAIVATSTIA*QQQPYPQQPQPFPQQPIPQQPQPFPQQPQPFPQQPIPQQPQPYPQQPQPFPQQPFPSQQPFPQQPRFWQQQPVLSQQQPCTQEQTPLLQEQQDQMLLQVQIPFVHPSILQQLNPCKVFLQQQCSPVAMSQRIARSQMLQQSSCHVLQQQCCQQLPQIPEQLRHEAVRAIVYSIVLQEQSLQLVQGVSQPQQQSQQQQVGQCSFQQPQPQQGQQQQVPQSVLLQPHQIAQLEATTSIALRTLPTMCSVNVPLYRIVPL**AIDTRVGV**					
QQEKVGQCSF	I6TRT2-C1	5.28	605.780	697.297 (y6, 1+)<br>825.392 (y7, 1+)<br>954.435 (y8, 1+)	28.7

GQPQQVPQSVF	I6TRT2-C2	4.70	607.812	577.298 (y5, 1+)<br>638.326 (b6, 1+)<br>863.437 (b8, 1+)	28.8
*MKTFLIFALLVIAATSTIA*QQQPYPQQPFQPQQPFPQQTIPQQPQPYPQQPQPYPQQPFPPQQEFPQQPPFWPQQPFPQQPPFGLQQRILSQQQPCTPQQTPLPQGQLYQTLLQLQIPYVHPSILQQLNPCKVFLQQQCSPVRMPQLIARLQMLQLSSCHVLQQQCCQQLPQISEQFRHEAIRAIVYSIFLQEQPQQSVQGVSQTQQQL**QQEKVGQCSF**QQPQPQQL**GQPQQVPQSVF**LQPHQIAQLEATTSIALRTLPRMCNVNVPLYDIMPPDFWH					
QQKPFPQQPPF	P06470-C1	5.11	671.351	726.393 (b6, 1+)<br>854.452 (b7, 1+)<br>982.511 (b8, 1+)	31.9
RHEAIRAIVY	P06470-C2	6.26	614.352	805.493 (y7, 1+)<br>934.536 (y8, 1+)<br>1071.595 (y9, 1+)	29.1
RGVGPSVGV	P06470-C3	3.39	414.240	554.305 (b6, 1+)<br>653.373 (b7, 1+)<br>710.394 (b8, 1+)	19.3
*MKTFLIFALLAIAATSTIA*QQQPFPQQPIPQQPQPYPQQPQPYPQQPFPPQQPFPQQPVPQQPQPYPQQPFPPQQPFPQQPPFW**QQKPFPQQPPF**GLQQPILSQQQPCTPQQTPLPQGQLYQTLLQLQIQYVHPSILQQLNPCKVFLQQQCSPVPVPQRIARSQMLQQSSCHVLQQQCCQQLPQIPEQF**RHEAIRAIVY**SIFLQEQPQQLVEGVSQPQQQLWPQQVGQCSFQQPQPQQVGQQQQVPQSAFLQPHQIAQLEATTSIALRTLPMMCSVNVPLYRIL**RGVGPSVGV**					
LPQKPFPVQQPF	Q40053-C1	5.98	713.399	907.541 (b8, 1+)<br>1035.599 (b9, 1+)<br>1163.658 (b10, 1+)	34.0
QPQQPFPQGSEQIIPQQPFPL	Q40053-C2	8.01	802.081	826.446 (y7, 1+)<br>939.530 (y8, 1+)<br>1095.522 (b10, 1+)	36.5
QPQPFPQQPQQPLPQPQQPF	Q40053-C3	6.37	785.734	951.468 (b8, 1+)<br>841.420 (y7, 1+)<br>1051.557 (y9, 1+)	35.7
IIPQQPQQPLPL	Q40053-C4	6.53	686.403	732.461 (y7, 1+)<br>933.515 (b8, 1+)<br>1143.652 (b10, 1+)	32.6
MKTFLTFVLLAMVMSIVTTARQLNPSSQELQSPQQSYLQQPYPQNPY**LPQKPFPVQQPF**HTPQQYFPYLPEELFPQYQIPTPLQPQQPFPQQPQQPLRPQQPFPWQPQQPFPQPQEPIPQQPQQPFPQQPQQPFPQQPQQIIFQQPQQSYPVQPQQPFPQPQPVPQQRPQQASPLQPQPQQASPL**QPQQPFPQGSEQIIPQQPFPLQPQPFPQQPQQPLPQPQQPF**RQQAEL**IIPQQPQQPLPL**QPHQPYTQQTIWSMV					
LPQQPFPVQQPF	Q41210-C1	6.97	713.380	808.435 (b7, 1+)<br>907.504 (b8, 1+)<br>1035.562 (b9, 1+)	34.0
QPQQPFPQQPQQPLPRPQQPFPW	Q41210-C2	7.15	932.145	1079.527 (b9, 1+)<br>1152.595 (y9, 1+)<br>1362.732 (y11, 1+)	42.7

QPQQPFPQPQQPIAHQPQQPF	Q41210-C3	5.54	822.749	1162.600 (y10, 1+)<br>951.468 (b8, 1+)<br>1102.553 (b19, 2+)	37.5
SQQPQQPFPL	Q41210-C4	5.86	585.301	697.326 (b6, 1+)<br>941.448 (b7, 1+)<br>826.446 (y6, 1+)	27.7
QPQQPFPQQPQQPFPQQPQQIIF	Q41210-C5	7.54	925.473	1098.594 (y9, 1+)<br>951.468 (b8, 1+)<br>1079.527 (b9, 1+)	42.4
MKTFLTFVLLAMVMSIVTTARQLNPSSQELQSPQQSYLQQPYPQNPY**LPQQPFPVQQPF**HTPQQYFPYLPEELSPQYQIPTPL**QPQQPFPQQPQQPLRPQQPFPW**QPQQPFPQPQ QPIPYQPQQPFNQQPQQIISQQPQQPFPQQPQQPFPQPQQPFPWQPQQPFPQPQQPFPLQPQQPFPW**QPQQPFPQPQQPIAHQPQQPF**SF**SQQPQQPFPLQPQQPFPQQPQQPFPQ QPQQIIF**QQPQQSYPVQPQQPFPQPQPVPQQRPQQASPLQPQQPFPQGSEQIIPQQPQQPFPLQPHQPYTQQTIWSMV					
IIPQQPQQPFPL	P17991-C1	6.95	703.396	826.446 (y7, 1+)<br>933.515 (b8, 1+)<br>1177.636 (b10, 1+)	33.5
FPQPQEPFPQQPQQPFPLQPQQPFPQQPQQPFPQPQQPFRQQAEL**IIPQQPQQPFPL**QPHQPYTQQTIWSMV					
EQQTEVPSKGGSF	I6TRS8-C1	3.12	697.333	679.341 (y7, 1+)<br>778.409 (y8, 1+)<br>907.452 (y9, 1+)	33.2
QSATSPQQPGQGQGQQETYPIATSPHQPGQW	I6TRS8-C2	5.00	1107.184	1221.601 (y11, 1+)<br>1318.654 (y12, 1+)<br>1246.583 (y23, 2+)	51.1
YPIATSPQQPGQGQQL	I6TRS8-C3	4.80	856.934	727.373 (y7, 1+)<br>858.436 (b8, 1+)<br>986.494 (b9, 1+)	41.0
GQGQQPGHGQQL	I6TRS8-C4	1.74	617.800	736.374 (y7, 1+)<br>1049.512 (y10, 1+)<br>847.381 (b9, 1+)	29.3
TQQKPGQGY	I6TRS8-C5	1.10	503.751	521.235 (y5, 1+)<br>649.330 (y6, 1+)<br>777.389 (y7, 1+)	23.7
HVSVEQPSASL	I6TRS8-C6	3.86	577.296	680.336 (b6, 1+)<br>864.421 (b8, 1+)<br>935.458 (b9, 1+)	27.3
*MAKRLVLFVAVIVALVALTTA*EREINGNNIFLDSRSRQLQCERELQESSLEACRRVVDQQLVGQLPWSTGLQMQCCQQLRDVSPECRPVALSQVVRQY**EQQTEVPSKGGSF**YPGGT APPLQQGGWWGTSVKWYYPDQTSSQQSWQGQQGYHQSVTSSQQPGQGQQGSYPGSTFPQQPGQGQQPGQRQPWSYPSATFPQQPGQGQGQQGYYPGATSLLQPGQGQQGPY**QSATS PQQPGQGQGQQETYPIATSPHQPGQW**QQPGQGQQGYYPSVTSPQQSGQGQQGYPSTTSPQQSGQGQQLGQGQQPGQGQQGYPSATFPQQPGQWQQGSYPSTTSPQQSGQGQQGYNP SGTSTQQPGQVQQLGQGQQGY**YPIATSPQQPGQGQQLGQGQQPGHGQQL**VQGQQQGQGQQGHYPSMTSPHQTGQGQKGYYPSAISPQQSGQGQQGYQPSGASSQGSVQGACQHSTS SPQQQAQGCQASSPKQGLGSLYYPSGAY**TQQKPGQGY**NPGGTSPLHQQGGGFGGLTTEQPQGGKQPFHCQQTTVSPHQGQQTTVSPHQGQQTTVSPHQGQQTTVSPHQGQQTTVS					

PHPGQQTTVSPHQGQQTTVSPHPGQQTTVSPHQGQQTTVSPHQGQQTTVSPHQGQQTTVSPHQGQQTTVSPHQGQQPGEQPCGFPGQQTTVSLHHGQQSNELYYGSPY**HVSVEQPSASL**KVAKAQQLAAQLPAMCRLEGGGGLLASQ					
VGVVTGVGGQ	I6TMV6-C1	3.78	436.746	518.257 (y6, 1+)<br>513.303 (b6, 1+)<br>612.372 (b7, 1+)	20.4
*MKILIILTILAMATTFATS*EMQVNPSVQVQPTQQQPYPESQQPFISQSPQQFPLPQQPFPQQPQQPFPQSQQQCLQQPQHQFPQPTQQFPQRPLLPFTHPFLPFPDQLLPQPPHQSFPQPPQSYPQPPLQPFPQPPQQKYPEQPQQPFPWQQPTIQLYLQQQLNSCKEFLLQQCRPVSLLSYLWSKILQQSSCRVMQQQCCLQLAQIPEQYKCTAIDSIVHAIFMQQGQRQGVQIVQQQPQPQQVGQCVLVQGQGVAQPQQLAQMEAIRTLVLQSVPSMCNFNVPPNCSTIKAPF**VGVVTGVGGQ**					
VQGRDVVQPQQL	TC131355-C1	3.87	683.876	754.421 (b7, 1+)<br>882.479 (b8, 1+)<br>1107.591 (b10, 1+)	32.5
*MKILIILIILAMATSFATS*EMQVNPSVQVQPTQQQTHPESQQPFIHHSQQQFPQPQQSFPQQPQQPFPQSQQPCLQQPQHQFPQPSQPFPQQPLQSFPRPFLPFPEQPLPQPPQESFPQPPQSYPQPPLQPFPQPPQESFPQPPQSYPQPLLQPFPQPPQQQYPEQPQQPFPRPPQEQFPNQPQQPFPWQQPSIQLYLQQHLNPCKEFLLQQCRPVSLVSYLWSKIVQQSNCQVMQEQCCLQLAQIPEQYKCTTIDSIVHAIFLQQGQRQGVQIVQQQPQPQEVGQCVL**VQGRDVVQPQQL**AQMEAIRSLVLQSVPAMCNFNVPPNCSTMRAPFFSLVNAGML					
QPQQQPQFPQQKPF	I6TEV2-C1	4.65	863.439	891.472 (y7, 1+)<br>1116.584 (y9, 1+)<br>982.474 (b8, 1+)	39.4
AQQQPSIEEQHQL	I6TEV2-C2	3.47	768.376	896.447 (y7, 1+)<br>983.479 (y8, 1+)<br>1080.532 (y8, 1+)	36.7
VLPQQQAQF	I6TEV2-C3	4.37	529.786	846.411 (y7, 1+)<br>694.388 (b6, 1+)<br>893.484 (b8, 1+)	25.0
MKIFLLFSLLGVATAITTTTMQFNPSGLELERPQQLFPQWQPLPQQPPFLQQEPEQPYPQQQPLPQQQPFPQQPQLPHQHQFPQQLPQQQFPQQMPL**QPQQQPQFPQQKPF**GQYQQPLTQQPYPQQQPL**AQQQPSIEEQHQL**NLCKEFLLQQCTLDEKVPLLQSVISFLRPHISQQNSCQLKRQQCCQQLANINEQSRCPAIQTIVHAIVMQQQQQQVQQQVDHGFVQSQLQQLGQGMPIQLQQQPGQAF**VLPQQQAQF**KVVGSLVIQTLPMLCNVHVPPYCSPFGSMATGSGGQ					

RT = retention time (min); Q1 = precursor ion *m/z* with charge, z; Q3 = product ion *m/z* with fragment ion assignment and charge, z; CE = collision energy (V).
